# An update on angiotensin-converting enzyme 2 structure/functions, polymorphism, and duplicitous nature in the pathophysiology of coronavirus disease 2019: Implications for vascular and coagulation disease associated with severe acute respiratory syndrome coronavirus infection

**DOI:** 10.3389/fmicb.2022.1042200

**Published:** 2022-11-28

**Authors:** Christian A. Devaux, Laurence Camoin-Jau

**Affiliations:** ^1^Aix-Marseille Université, IRD, APHM, MEPHI, IHU–Méditerranée Infection, Marseille, France; ^2^Center National de la Recherche Scientifique, Marseille, France; ^3^Laboratoire d’Hématologie, Hôpital de La Timone, APHM, Boulevard Jean-Moulin, Marseille, France

**Keywords:** ACE2, renin-angiotensin system, hypertension, coagulation, coronavirus—COVID-19, therapy

## Abstract

It has been known for many years that the angiotensin-converting enzyme 2 (ACE2) is a cell surface enzyme involved in the regulation of blood pressure. More recently, it was proven that the severe acute respiratory syndrome coronavirus (SARS-CoV-2) interacts with ACE2 to enter susceptible human cells. This functional duality of ACE2 tends to explain why this molecule plays such an important role in the clinical manifestations of coronavirus disease 2019 (COVID-19). At the very start of the pandemic, a publication from our Institute (entitled “ACE2 receptor polymorphism: susceptibility to SARS-CoV-2, hypertension, multi-organ failure, and COVID-19 disease outcome”), was one of the first reviews linking COVID-19 to the duplicitous nature of ACE2. However, even given that COVID-19 pathophysiology may be driven by an imbalance in the renin-angiotensin system (RAS), we were still far from understanding the complexity of the mechanisms which are controlled by ACE2 in different cell types. To gain insight into the physiopathology of SARS-CoV-2 infection, it is essential to consider the polymorphism and expression levels of the *ACE2* gene (including its alternative isoforms). Over the past 2 years, an impressive amount of new results have come to shed light on the role of ACE2 in the pathophysiology of COVID-19, requiring us to update our analysis. Genetic linkage studies have been reported that highlight a relationship between ACE2 genetic variants and the risk of developing hypertension. Currently, many research efforts are being undertaken to understand the links between ACE2 polymorphism and the severity of COVID-19. In this review, we update the state of knowledge on the polymorphism of ACE2 and its consequences on the susceptibility of individuals to SARS-CoV-2. We also discuss the link between the increase of angiotensin II levels among SARS-CoV-2-infected patients and the development of a cytokine storm associated microvascular injury and obstructive thrombo-inflammatory syndrome, which represent the primary causes of severe forms of COVID-19 and lethality. Finally, we summarize the therapeutic strategies aimed at preventing the severe forms of COVID-19 that target ACE2. Changing paradigms may help improve patients’ therapy.

## Introduction

Present in a large number of tissues, including endothelial cells of the arteries, arterioles, and venules of the heart and kidney, angiotensin-converting enzyme 2 (ACE2) is a fascinating molecule which plays a crucial role in maintaining blood pressure homeostasis. ACE2 is only one of the actors in a complex biological network known as the renin-angiotensin system (RAS). ACE2 mainly exerts its functions by regulating the ratio of two major mediators: angiotensin II (Ang II) and angiotensin-[1–7; Ang-(1–7)]. Ang II synthesis is catalyzed by angiotensin-converting enzyme (ACE) while Ang-(1–7) is obtained after hydrolysis of Ang II by ACE2. Ang-(1–7) can also be generated from Ang-(1–9) formed after the action of ACE2 on Ang I by the action of ACE itself. Despite their contrasting physiological functions, the ACE2 is considered to have evolved through ACE gene duplication and exhibits 42% amino acid homology with ACE (Donoghue et al., 2000; Turner and Hooper, 2002; Towler et al., 2004).

Besides being widely studied in cardiology, ACE2 became attractive for other fields of medical sciences and, particularly, virology (Devaux et al., 2020). In 2003 a novel coronavirus infecting humans, the severe acute respiratory syndrome coronavirus (SARS-CoV, provisionally renamed SARS-CoV-1) emerged in Asia, causing an outbreak of severe pneumopathy (Ksiazek et al., 2003; Marra et al., 2003; Rota et al., 2003). ACE2 was demonstrated to be the cellular receptor for SARS-CoV-1, as it had been previously reported for another coronaviruses infecting humans, HCoV-NL63, a coronavirus causing the common winter cold (Hofmann et al., 2005; Li et al., 2007; Ge et al., 2013; Graham et al., 2013). In 2019, new cases of severe pneumopathy were reported in China, with the disease being characterized by a multiple organ dysfunction syndrome (MODS) as well as acute respiratory distress syndrome (ARDS) sometimes requiring the need for ventilation or extracorporeal membrane oxygenation (ECMO). The severe forms of the disease lead to death in ∼ 0.5–2.5% of cases, with a high fatality risk increasing with age and the existence of underlying comorbidities (Huang et al., 2020; Zhou et al., 2020; Zhu et al., 2020). Under chest computerized tomography (CT) scans, the majority of patients show bilateral ground glass-like opacities and subsegmental areas of consolidation indicative of pneumonia. This disease was later defined as COVID-19, the aetiological agent of which was found to be a new human coronavirus named severe acute respiratory syndrome coronavirus (SARS-CoV-2). Although not highly symptomatic for the majority of those infected, the virus has spread worlwide causing more than 6 million deaths for ∼603 million reported cases of infections (World Health Organization COVID-19 Dashboard on 6 September 2022; https://covid19.who.int/). SARS-CoV-2 shares 79.5% nucleotide identity with SARS-CoV-1, and both these Sarbecoviruses isolated from humans are genetically close to coronaviruses circulating in wildlife (Ge et al., 2013; Afelt et al., 2018; Wang et al., 2020; Zhou et al., 2020; Frutos et al., 2021). Once SARS-CoV-2 was characterized, the search for its cellular receptor became a priority. Due to the sequence similarity between SARS-CoV-1 and SARS-CoV-2, studies quickly focused on ACE2 and the role of this molecule as a viral entry receptor was demonstrated (Qiu et al., 2020; Yan et al., 2020).

Due to the central role played by ACE2 in maintaining blood pressure homeostasis, the objective of this work is to review the state of knowledge regarding the possible imbalance of the RAS in the context of a SARS-CoV-2 infection and to highlight the role of ACE2 in SARS-CoV-2 infection and replication, as well as its contribution in the severity of COVID-19.

## The renin-angiotensin system: A molecular network which regulates blood pressure homeostasis and ion-fluid balance

In humans and other mammals, intravascular RAS plays a key role in maintaining blood pressure homeostasis as well as fluid and salt balance, while tissue RAS is mainly involved in the pathogenesis of inflammatory diseases (Paul et al., 2006; Greenberg, 2008; de Kloet et al., 2010). The kidneys, as a sensor of ion fluid balance and producer of renin, play a fundamental role in the long-term control of arterial pressure (Tigerstedt and Bergman, 1898; Phillips and Schmidt-Ott, 1999; Yim and Yoo, 2008; Prieto et al., 2011; Gonzalez et al., 2017). Active renin is secreted into the blood circulation in response to hypotension or hypernatremia. Upon activation of the juxtaglomerular apparatus of the kidneys’ afferent arterioles, proteases (proconvertase 1, cathepsin B) catalyze the removal of the 20-amino-acid terminal prosegment of prorenin to produce a polypeptide composed of 297 amino-acids (Davis and Freeman, 1976; Hadman et al., 1984; Cohen-Haguenauer et al., 1989; Sealey and Rubattu, 1989; Neves et al., 1996; Muller et al., 1999). The active form of renin cleaves the alpha-globulin angiotensinogen (formerly angiotonin, a 118-amino-acid-long polypeptide), giving rise to angiotensin I (Ang I), the N-terminal decapeptide of angiotensinogen (Goldblatt et al., 1934; Page and Helmer, 1940; James and Sielecki, 1985). The conversion of Ang I (Asp-Arg-Val-Tyr-Ile-His-Pro-Phe-His-Leu) to the octapeptide Ang II (Asp-Arg-Val-Tyr-Ile-His-Pro-Phe), requires the cleavage of its C-terminal dipeptide catalyzed by ACE (provisionally named ACE1) expressed at the endothelial surface of the blood vessels, epithelium of the lungs and upper respiratory system (Skeggs et al., 1956; Crisan and Carr, 2000; Wakahara et al., 2007). The vasoconstrictor octapeptide Ang II was evidenced to be a substrate for ACE2, which acts as an essential factor in the RAS pathway homeostasis. By removing a single residue phenylalanine (Phe) from Ang II, the membrane form of ACE2 (mACE2) plays a central role in the synthesis of the cardiovascular protective heptapeptide Ang-(1–7) that acts by limiting the adverse vasoconstrictor and profibrotic effects of Ang II and reduces the oxidative stress of Ang II on endothelial arteries (Crackower et al., 2002; Pena Silva et al., 2012). ACE2 can also catalyze the conversion of Ang I to Ang-(1–9) by removing the C-terminal leucine (Leu) residue of Ang I, but with a catalytic efficiency ∼ 400-fold lower than the hydrolysis of Ang II to produce Ang-(1–7). Besides Ang II and Ang I, ACE2 can cleave several other substrates including des-Arg9-bradykinin (DABK), apelin-13, and dynorphin A-(1–13; Skidgel and Erdos, 1987; Ferrario et al., 1997; Vickers et al., 2002; Oudit et al., 2003) In addition to its membrane form, ACE2 can be found in a soluble form (sACE2) and increasing sACE2 has been reported in patients with cardiomyopathies and heart failure (Epelman et al., 2008). In patients with aortic stenosis, increasing levels of sACE2 associated with reduced myocardial *ACE2* gene expression and severe myocardial fibrosis is considered as a death risk biomaker (Rajagopal et al., 2010). Thus, increased sACE2 plasma levels have been associated with heart failure, cardiovascular disease, and cardiac remodeling (Epelman et al., 2009; Sama et al., 2020; Garcia-Escobar et al., 2021). Using animal models, it was shown that knocking out (KO) of the *ACE2* gene results in increased levels of Ang II, followed by vasoconstriction reducing coronary blood flow and leading to cardiac dysfunction (Danilczyk et al., 2003). The expression of mACE2 in the kidneys and heart is influenced by salt rich and/or glucose-rich diets, and can be correlated with pathological disorders (Reich et al., 2008; Lavrentyev and Malik, 2009; Bernardi et al., 2012; Wysocki et al., 2013). In the respiratory tract, DABK is a substrate of mACE2 and a decrease in ACE2 could lead to an increase in vascular permeability and fluid extravasation (Chung et al., 2020). Using a mouse animal model, it was found that loss of ACE2 led to activation of the DABK/braddykinin receptor B1 (BKB1R) axis associated with release of proinflammatory chemokines (e.g., CXCL5, MIP2, and TNFα) and increase in neutrophil infiltration (Sodhi et al., 2017).

Resulting from the cleavage of Ang II by the mACE2 protease, Ang-(1–7) exhibits vasodilatory, anti-proliferative, anti-inflammatory, and antifibrotic effects *via* the G protein-coupled receptor (GPGR) known as Mas 1 (Santos et al., 2003, 2018; Simoes e Silva et al., 2013; Patel et al., 2016; Karnik et al., 2017; Bader et al., 2018). However, biochemical studies have failed to demonstrate a direct interaction between Ang-(1–7) and Mas1 (Gaidarov et al., 2018). In addition to mACE2, several peptidases, including vascular endothelium prolyl peptidases, neprilysin (NEP), and smooth muscle thimet oligopeptidase, can produce Ang-(1–7; Chappell, 2019). NEP and thimet oligopeptides produce Ang-(1–7) directly from Ang I. Ang-(1–7) has been shown to potentiate bradykinin (BK 1–9), a potent vasodilator of the kinin system which mediates its effects through the B2 receptor (BKB2R) abundant in vascular tissue (Jackman et al., 2002). ACE2 overexpression and Ang-(1–7) infusion have beneficial effects on atherosclerosis, whereas ACE2 deficiency accentuates vascular atherosclerosis in animal models (Dong et al., 2008; Thomas et al., 2010; Yang et al., 2013). The up-regulation of the ACE2/Ang-(1–7)/MasR axis promotes the expression of E-cadherin (E-cad) adhesion molecules by suppressing the PAK1/NF-κB/Snail1 pathway (Yu et al., 2016). Moreover, Ang-(1–7) can exert cerebroprotective functions in endothelin-1-induced ischaemic stroke (Mecca et al., 2011).

For many years, it has been known that there is cross-talk between insulin and the RAS, providing possible links between hypertension, obesity, and diabetes (Alderman et al., 1991; Frederich et al., 1992; Velloso et al., 1996; Boustany et al., 2004; Schmieder et al., 2007). Moreover, a low expression of ACE2 mRNA or protein is associated with an increase in AngII levels, hypertension, diabetes and heart disease (Crackower et al., 2002; Diez-Freire et al., 2006; Tikellis et al., 2012; Velkoska et al., 2016).Interestingly, these diseases are the major comorbidities in the severe forms of COVID-19 (Bavishi et al., 2020). The occurrence of specific comorbidities associated with an RAS imbalance could be decisive for the clinical outcome of COVID-19 (Devaux et al., 2020; Rysz et al., 2021).

## RAS imbalance and overproduction of harmful Ang II

Clinical investigations have provided convincing evidence that RAS imbalance is capable of stimulating atherosclerosis, which ultimately lead to the rupture of atherosclerosis plaques and thrombosis (Schmidt-Ott et al., 2000; Jacoby and Rader, 2003; Verdecchia et al., 2008). Ang II is the main harmful effector molecule synthesized in excess in situations of RAS imbalance. Ang II, inactivates the vasodilator bradykinin and can control the ion-fluid balance by acting on the adrenal cortex to stimulate the release of aldosterone, leading to sodium and water retention (Jaspard et al., 1993; Brewster and Perazella, 2004; Xue et al., 2012; Aroor et al., 2016; Nishimura, 2017). The action of Ang II (proximal tubule) and aldosterone (collecting duct) are complementary to influence sodium reabsorption across the nephron (Gurley et al., 2011). Thereby, Ang II functions as a powerful regulator of vascular tone and intravascular volume. Increased circulating levels of Ang II is associated with vasoconstriction and hypertension and accelerates thrombosis in arterioles by activating the coagulation cascade and the platelet-derived growth factor (PDGF; Gustafsson and Holstein-Rathlou, 1999; Heeneman et al., 2000; Senchenkova et al., 2010, 2014; Singh and Karnik, 2016; Samavati and Uhal, 2020). It also induces hypertrophy of vascular smooth muscle cells (Berk et al., 1989; Griendling et al., 1997; Funakoshi et al., 2002). Ang II can also exert tissue-specific actions, such as neurotransmission inducing adipocytes growth in adipose tissues (Li and Ferguson, 1993; Massiéra et al., 2001).

These multiple effects of Ang II are obtained through its ability to bind to Ang II type I and type II receptors (AT1R and AT2R, respectively) expressed in arterioles and several organs including the kidney, pancreas, heart, and the brain. The AT1R, a 359-amino-acids protein spanning cell membrane, and AT2R have a 34% nucleic acid sequence homology (Arendse et al., 2019). Ang II can bind to both to AT1R and AT2R, which are receptors with opposite effects (i.e., AT1R mediates vasoconstriction, inflammation and fibrosis while AT2R mediates opposite effects). AT2R is poorly expressed compared to AT1R, which causes the Ang II to primarily exhibit an effect through AT1R (Murphy et al., 1991; de Gasparo et al., 2000; Forrester et al., 2018; Furuhashi et al., 2020). The activation of AT1R by Ang II is transient and associated with the phosphorylation of the receptor by kinases, including PKC and GRKs. The phosphorylated AT1R is internalized through a mechanism that involves β-arrestin 2, the adaptor protein complex 2 (APC2), clathrin, and intersectin 2 (AbdAlla et al., 2000; de Gasparo et al., 2000; Gáborik et al., 2001). These AT1R-mediated signals lead to overexpression of the prorenin receptor (PRR), thereby increasing renin activity and contributing to the local accumulation of Ang II, fibrosis, and hypertension (Nguyen et al., 2002; Advani et al., 2009; Peng et al., 2013; Wang et al., 2014; Xu et al., 2016; Ichihara and Yatabe, 2019). At the opposite, Ang II also exerts a negative feedback signaling on juxtaglomerular cells that reduces the *REN* gene transcription and renal renin secretion (Naftilan and Oparil, 1978).

The interaction of Ang II with AT1R functions as a pluripotent mediator to enhance oxidative injury by reactive oxygen species (ROS), and endothelial injury by inhibiting nitric oxide (NO) synthesis. Ang II is a potent activator of NADPH oxidase and an inducer of ROS (Garrido and Griendling, 2009). Interestingly, CHOP−/− mice are protected from Ang II-induced NADPH oxidase activation, hypertension, and cardiovascular disease (Kassan et al., 2016). This is consistent with the observation that Ang II increases the transcription of the *CHOP* and *ATF4* genes (Kassan et al., 2012; Spitler and Webb, 2014; Takayanagi et al., 2015). Activation of AT1R by Ang II also induces various signaling pathways, including G-protein-coupled receptors, PKC, serine/threonine kinase, serine tyrosine kinases, ERK/JNK activation, leading to proinflammatory responses characterized by the synthesis of IL-6, TNFα, and other cytokines (Sadoshima et al., 1995; Han et al., 1999; Nataraj et al., 1999; Ruiz-Ortega et al., 2001; Watanabe et al., 2005; Luther et al., 2006; Rushworth et al., 2008; Dikalov and Nazarewicz, 2013). Furthermore, Ang II activates the flow of neutrophils and macrophages to the affected tissues and inhibits the production of NO, leading to vascular injury (Nabah et al., 2004).

## ACE2 tissue distribution in human

**Figure 1:**
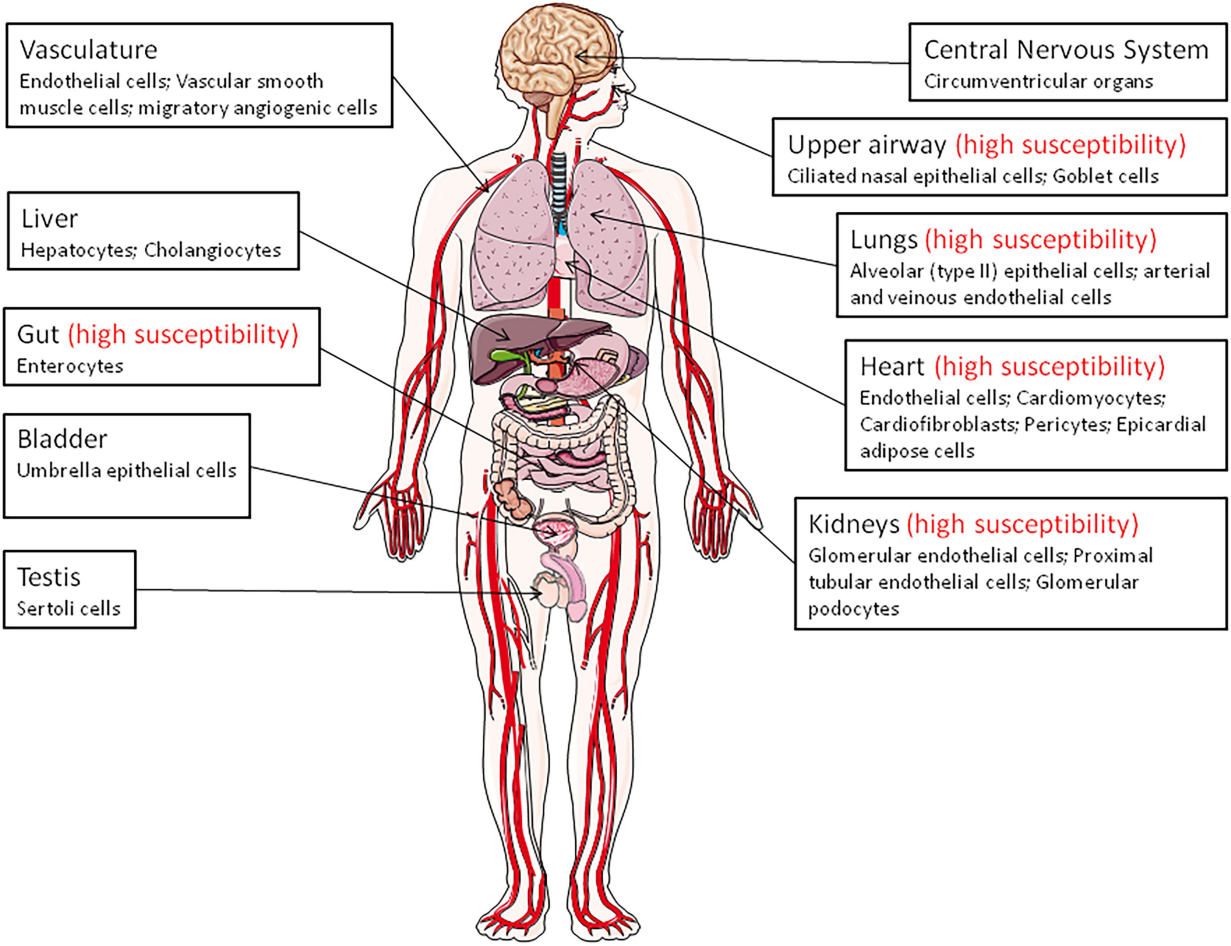
Angiotensin-converting enzyme 2 (ACE2) expression throughout the body (the main ACE2^+^ target cells are indicated). The organs vulnerability to SARS-CoV-2 infection is also indicated (high susceptibility).

Angiotensin-converting enzyme 2 is expressed in virtually all organs with higher levels in capillary rich organs such as the lungs, heart, or kidneys (Donoghue et al., 2000; Tipnis et al., 2000; Ferrario and Varagic, 2010; Tikellis and Thomas, 2012; Figure 1). A study of ACE2 mRNA and protein in more than 150 cell types concluded that ACE2 is mainly observed in enterocytes, renal tubules, the gallbladder, cardiomyocytes, male reproductive cells, placental trophoblasts, ductal cells, eyes, and the vasculature. In the respiratory system, its expression was limited to a subset of cells (Hikmet et al., 2020).

Remarkably, in the upper airway, goblet and ciliated cells show the highest expression of ACE2 and are thought to play a major role in human infection with SARS-CoV-2. The expression of the mACE2 protein is highest within regions of the sinonasal cavity and pulmonary alveoli and in the lung parenchyma (Descamps et al., 2020; Ortiz et al., 2020). In normal human lungs, the mACE2 protein is found on a very small subset of alveolar type II epithelial lung cells (Ortiz et al., 2020; Delorey et al., 2021). Alveolar epithelial type II cells (which represent ∼5% of the alveoli and serves at stem cells to generate type I alveolar epithelial cells), are thought to be a main target for SARS-CoV-2 in the respiratory tract and, consequently, can be destroyed during viral replication (Barkauskas et al., 2013). However, ACE2-positive cells are more abundant in the nasal mucosa than in the bronchus (Hikmet et al., 2020). Moreover, the mACE2 peptidase is also expressed in the arterial and venous endothelial cells present in abundance in the lungs and arterial smooth muscles (Hamming et al., 2004). Expression of ACE2 was found to be drastically increased in airway epithelial cells 24 h after SARS-CoV-1 infection (Li et al., 2020). In COVID-19 related ARDS, ACE2 was found to be upregulated in endothelial cells, but not in type II alveolar epithelial lung cells (Gerard et al., 2021).

The expression of ACE2 in the heart is higher than in the lungs and ACE2 is found in the endothelial cells of coronary arteries, arterioles, venules, and capillaries (Danilczyk et al., 2003; Robinson et al., 2020). The mACE2 is strongly expressed in cardiomyocytes, endothelial cells, cardiac fibroblasts, vascular smooth muscle cells, and was also found in cardiac pericytes, which play crucial role in the microvasculature and may be the target for SARS-CoV-2 (Chen et al., 2020; Hikmet et al., 2020). Patients with heart failure show a significant increase in ACE2 mRNA expression (Goulter et al., 2004), suggesting that *ACE2* gene overexpression may explain why heart dysfunction is found within the list of COVID-19 comorbidities. In a rat model of diabetic cardiomyopathy, the overexpression of ACE2 attenuates cardiac hypertrophy, myocardial fibrosis, and dysfunction induced by diabetes (Dong et al., 2012). Post-mortem examinations of endomyocardial biopsies from COVID-19 patients highlighted the presence of SARS-CoV-2 in the myocardium (Lindner et al., 2020; Marchiano et al., 2021).

In the kidneys, ACE2 is expressed in the proximal tubule cells, epithelial cells of the Bowman’s capsule, endothelial cells, mesengial cells (glomerulus central area), glomerular podocytes, proximal cell brush border, and cells from the collecting ducts (Aragao et al., 2011; Hikmet et al., 2020; Martinez-Rojas et al., 2020). Patients with diabetic or hypertensive nephropathy had lower glomerular ACE2 expression compared to healthy controls (Mizuiri et al., 2008; Wysocki et al., 2013). Between 3 and 10% of COVID-19 patients have abnormal renal function (diagnosed with elevated creatinine or urea nitrogen), and 7% experienced acute renal injury (Fan et al., 2021). In the pancreas, ACE2 plays a major glycemia-protective role (Pedersen et al., 2013). In testis, the Sertoli cells, which protect germ cells by forming blood-testis barrier, have a high expression of mACE2, suggesting that SARS-CoV-2 might cause reproductive disorders in infected patients (Shen et al., 2020; Fan et al., 2021).

A high expression of ACE2 was reported in the epithelial cells of the oral mucosa. This is rarely seen in esophageal mucosa (mainly composed of squamous epithelial cells) and is abundantly expressed in the glandular cells of the gastric, duodenal, and rectal epithelia, possibly contributing to the oral transmission of SARS-CoV-2 and then to viral spreading into the gastrointestinal tract, a major target for the virus (Lamers et al., 2020; Xu et al., 2020; Devaux et al., 2021a; Osman et al., 2022). mACE2 is highly expressed thorough the ileum where it may cleave circulating Ang II in the mesenteric arterial blood into Ang-(1–7), which is destined for portal circulation and the liver. The mACE2 also exerts RAS-independent functions in the gastrointestinal tract through cleaving carboxy-terminal amino acids from nutrient proteins and by acting as a chaperon for the expression of the B^0^AT1 amino acid transporter (Crackower et al., 2002; Camargo et al., 2009; Singer and Camargo, 2011; Fairweather et al., 2012; Hashimoto et al., 2012; Vuille-Dit-Bille et al., 2015; Wang et al., 2015). The mACE2 regulates the gut homeostasis, microbiota composition, the expression of antimicrobial peptides (Reg3γ, α-defensin, such as HD5 and HD6, β-defensin, and lysozyme; Singer et al., 2012; Perlot and Penninger, 2013; Ferrand et al., 2019). This probably explains the diarrhea that is sometimes observed in SARSCoV-2 patients, and supports the use of antibiotic treatment in COVID-19 patients. In addition, it was reported that HD5 secreted by intestinal Paneth cells, interacts with ACE2 (Wang et al., 2020), suggesting that the presence of HD5 in abundance in the ileal fluid may compete with SARS-CoV-2 to bind to ACE2. The infection of Caco2 cells by SARS-CoV-2 was found to be significantly reduced when cultured in the presence of HD5 and this effect was confirmed on intestinal and lung epithelial cells and for different SARS-CoV-2 variants (Wang et al., 2020; Xu et al., 2021). Although the ACE2 regulation of gut homeostasis was considered to be RAS-independent, α-defensins expression has also been associated with atherosclerosis, being involved in the lipoprotein metabolism in the vessel wall and inhibiting fibrinolysis (Kougias et al., 2005; Nassar et al., 2007; Abdeen et al., 2021).

## Structure of the human ACE2 protein

**Figure 2:**
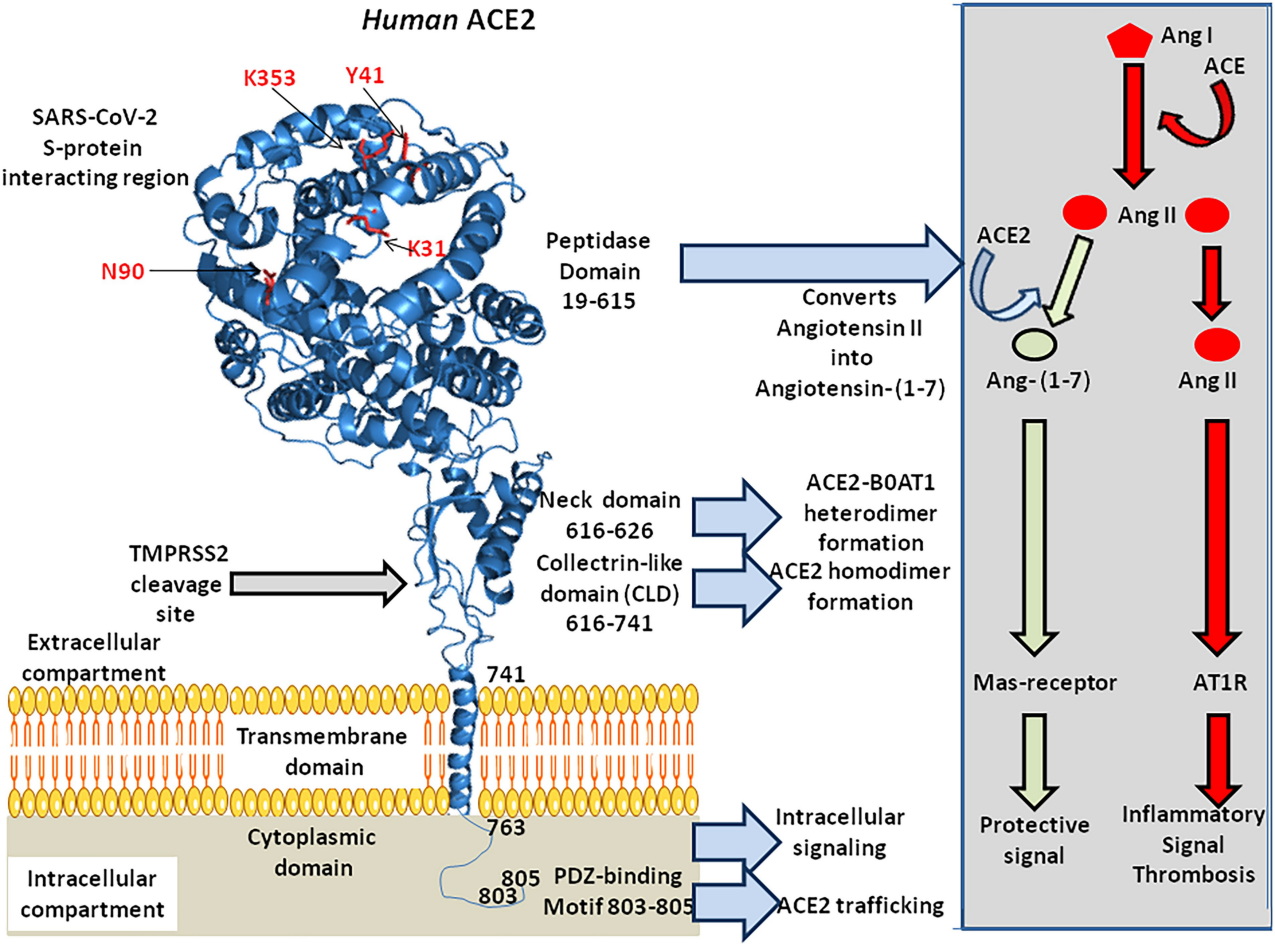
3-D model of ACE2 protein structure according to the published data PDB: 6M1D by Yan and colleagues in 2020. ACE2 is composed of 805 amino acids and characterized by an N-terminal signal peptide of 17 amino acid residues, a peptidase domain (PD; residues 19–615) with its HEXXH zinc binding metalloprotease motif, a C-terminal collectrin-like domain (CLD; residues 616–741) that includes a ferredoxin-like fold “neck” domain (616–626), that end with a hydrophobic transmembrane hydrophobic helix region (741–763) followed by an intracellular segment of 43 amino acid residues. No information is available regarding the 3-D structure of the ACE2 cytoplasmic tail (763–805), which was drawn to appear on the diagram. Some of the amino acids which are considered important for SARS-CoV-2 interaction are located in the 3-D model (amino acids in red). Arg_652_, Arg_708_, and Arg_710_ (not shown) are the active residues for ACE2-TMPRSS2 docking. The function of each domain is indicated on the middle right side of the figure. The biochemical pathway of the RAS and the beneficial ACE2/Ang-(1–7) arm of RAS are summarized in the right box.

The *ACE2* gene encodes a type I transmembrane glycoprotein of ∼ 100 kDa composed of 805 amino acids(Figure 2; Marian, 2013; Gheblawi et al., 2020), including six amino acids (Asn_53_, Asn_90_, Asn_103_, Asn_322_, Asn_432_, and Asn_546_), which can potentially be N-glycosylated (Lubbe et al., 2020). This metalloprotease resembles a chimera molecule composed of a single ACE-like catalytic ectodomain (41.8% sequence homology with the amino domain of ACE) fused to a collectrin-like domain (48% homology with collectrin; Donoghue et al., 2000; Zhang et al., 2001). The functional domains of ACE2 include: (i) a N-terminal signal peptide region of 17 amino acid residues; (ii) a peptidase domain (PD; amino acids 19–615) with its zinc binding metalloprotease motif (catalytic domain; amino acids 374–378); (iii) a C-terminal collectrin-like domain (CLD; amino acids 616–768 acting as a regulator of renal amino acid transport and insulin exocytosis), containing a ferredoxin-like fold “neck” domain (amino acids 615–726); and (iv) an hydrophobic transmembrane hydrophobic helix region of 22 amino acids followed by an intracellular cytoplasmic tail of 43 amino acids (Donoghue et al., 2000; Zhang et al., 2001; Cerdà-Costa and Gomis-Rüth, 2014). The C-terminal segment of mACE2 contains a PDZ-binding motif (amino acids 803–805) Thr_803_-Ser_804_-Phe_805_ (TSF_COOH_) targeting protein-interacting domains from proteins (SNX27, SHANK3, MAST2, and NHERF2) involved in protein trafficking (Caillet-Saguy and Wolf, 2021; Kliche et al., 2021).

The mACE2 functions predominantly as a monocarboxypeptidase, with a substrate preference for hydrolysis between a proline and a hydrophobic or basic C-terminal residue (Turner and Hooper, 2002). The catalytic domain of mACE2 consists of two subdomains (subdomains 1 and 2) forming the two sides of a long deep cleft bridged together by a hinge region. Upon substrate binding, the two catalytic subdomains undergo a hinge-bending movement and form a binding cavity required to initiate substrate hydrolysis (Towler et al., 2004). The His-Glu-X-X-His motif (or HEXXH motif where X is any amino acid), coordinates a catalytic zinc ion, characteristic of zinc-dependent metalloproteases. The zinc is co-ordinated by His_374_, His_378_, Glu_402_, and one water molecule in the subdomain 1, whereas a chloride ion is co-ordinated by Arg_169_, Trp_477_, and Lys_481_ in the subdomain 2. The Arg_514_ of mACE2 is considered as a residue critical for substrate selectivity (Luther et al., 2006).

Both the PD and neck domains of mACE2 contribute to dimerization, whereas each B^0^AT1 interacts with the neck and TM helix in the adjacent mACE2 (Yan et al., 2020). Complexes of mACE2/B^0^AT1 heterodimers have been evidenced at the intestinal apical membrane but did not occur in lung pneumocytes. Steric hindrance to the B^0^AT1 binding site on mACE2 or down-regulation of mACE2 due to the presence of SARS-CoV-2 is likely to display impaired intestinal tryptophan uptake (Devaux et al., 2021a).

Finally, the Arg_652_ of ACE2 is a target for the catalytic site of proteases ADAM17 and TMPRSS2, which leads to the shedding of a soluble form of ACE2 (sACE2; Heurich et al., 2014; Lanjanian et al., 2021).

## The human *ACE2* gene variant mRNAs

The prototype human *ACE2* cDNA (or *ACHE* for angiotensin-converting enzyme homolog) was cloned more than 2 decades ago from a human cardiac left ventricle cDNA library and a lymphoma cDNA library (Donoghue et al., 2000; Ferrario and Varagic, 2010). The *ACE2* gene, which contains 20 introns and 19 exons maps to chromosome Xp22 and spans 39.98 kb of genomic DNA (Turner and Hooper, 2002). Two isoforms of ACE2 with 18 or 19 exons (v1 and v2) that encode the same protein (805 amino acids) have been described, as well as three other smaller variants: x1–x3 (Chen et al., 2020; Khayat et al., 2020). ACE2 shows similarities with the ACE gene located at chromosome 17q23 (Hubert et al., 1991). Although *ACE2* is one of the genes escaping X chromosome inactivation, there is evidence of sex bias (Tukiainen et al., 2017; Cai, 2020; Gay et al., 2021). Indeed, there is a plausible mechanism of androgen-induced expression of ACE2 that contributes to increased susceptibility or severity of COVID-19 in males (Baratchian et al., 2021). The tissue levels of mACE2 represent equilibrium between transcription/translation of mACE2 and shedding rate of sACE2. It was reported that a positive relationship exists between renin and sACE2 levels in male and female subjects, and between sACE2 levels and body mass index (BMI) in males, with possible implication for COVID-19 (Jehpsson et al., 2021). Variations in mACE2 with age were first demonstrated using animal models (Xie et al., 2006). A negative association between age and sACE2 plasma concentrations in people above the age of 55 year-old, was reported (AlGhatrif et al., 2021). The mACE2 deficiency is considered to be linked to cardiovascular disease and diabetes, suggesting that mACE2 deficiency may increase the risk of developing severe COVID-19 (Oudit and Pfeffer, 2020; Verdecchia et al., 2020; Wang et al., 2020).

**Figure 3:**
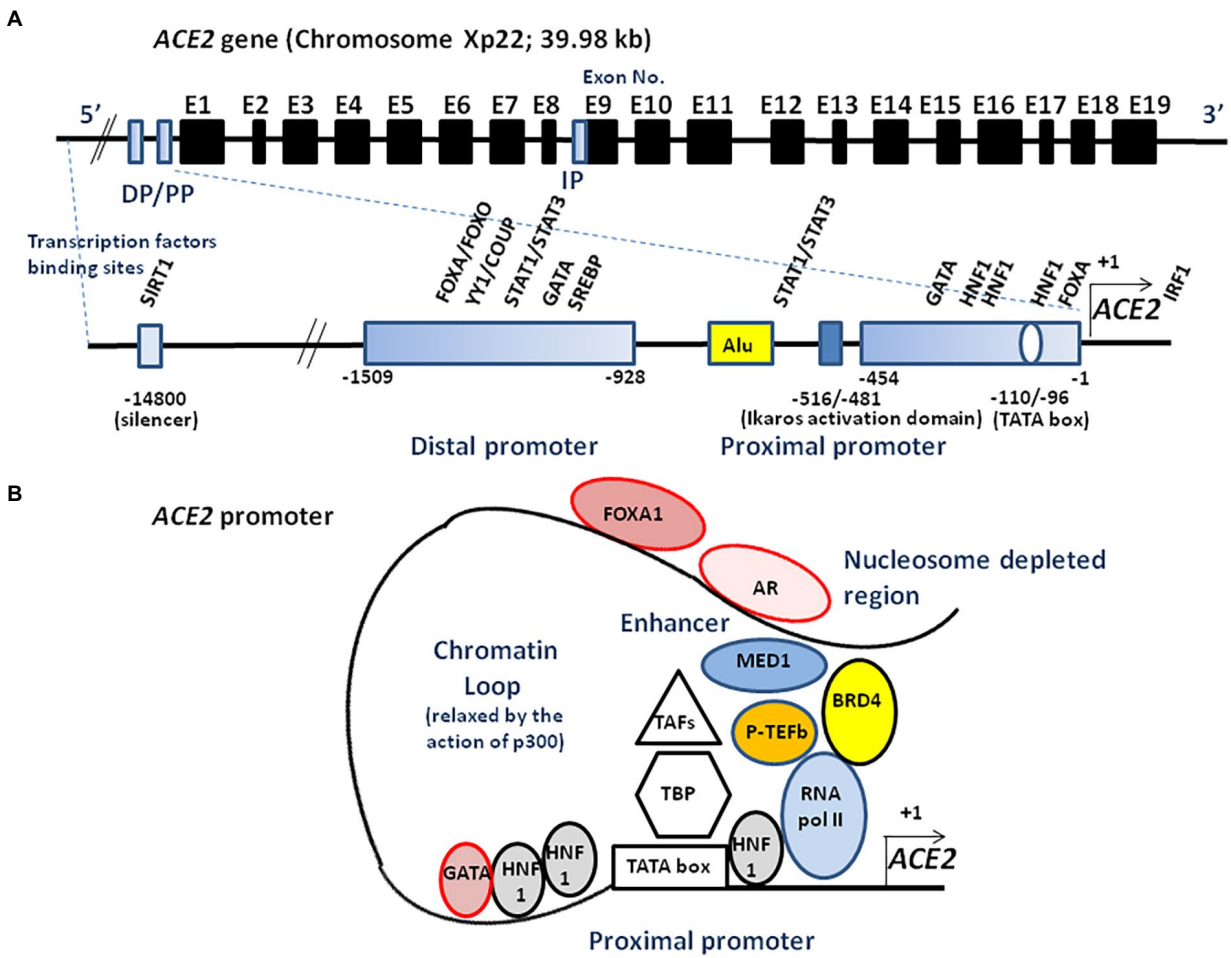
Schematic illustration of *ACE2* transcriptional regulation. **(A)** A schematic diagram of the *ACE2* gene structure (upper panel). The known exons (E1–E19) are depicted as black boxes. The location of the distal promotor (DP) and proximal promoter (PP) are depicted as blue boxes. The ACE2 gene can encode several transcript leading to several isoforms. An internal promoter (IP) is thought to activate the transcription of an mRNA encoding a short isoform of ACE2 which lacks the SARS-CoV-2 binding site. The 5′ region upstream of the *ACE2* gene contains two promoters (proximal and distal) separated by a repetitive Alu element (lower panel). The transcription of full-length ACE2 is initiated from either the proximal or distal promoter with tissue-specific differences in their usage. Transcription factors binding to the proximal and the distal upstream promoter regions are indicated. Ang II is likely to regulate the ACE2 expression through the Ikaros activation domain. Truncated ACE2 forms (e.g., *dACE2*) can also be expressed. **(B)** *ACE2* transcriptome. AR binds to the enhancer element of the ACE2 gene, connecting the regulatory circuit between the enhanceosome complex (comprising MED1, BRD4, etc.) and the promoter-bound RNA polymerase machinery to activate gene expression. P-TEFb, positive transcription elongation factor; TBP, TATA-binding protein; TAFs, TBP-associated factors; FOXA1, forkhead box A1; BRD4, bromodomain-containing protein 4; MED1, mediator complex subunit 1; SREBP, sterol regulatory element binding protein; and SIRT1, silent information regulator T1.

The transcription of full-length ACE2 (2,721 bp mRNA) is initiated from either a proximal or a distal promoter with tissue-specific differences in their usage (Itoyama et al., 2005; Fan et al., 2021). The proximal site contains a TATA box motif at position-110/−96 of the transcription start site and a GATA motif and two HNF1 binding sites at position-165/−131. The distal site contains YY1/COUP, C/EBPβ, and STAT/FOXA motifs. Site-directed mutagenesis of the human *ACE2* promoter region from position −2069 to +20, has enabled the identification of an activating domain in the −516 to −481 region (Kuan et al., 2011) and a potential binding site, ATTTGGA, homologous to that of an Ikaros-like binding domain which can be regulated by the levels of Ang II. It has also been reported that the NAD + -dependent deacetylase silent information regulator T1 (SIRT1 known for its ability to deacetylate proteins such as p53 and forkhead box O), binds to the *ACE2* promoter and regulates *ACE2* gene expression under condition of energy stress which increase AMP-activated protein kinase, while IL-1β treatment decreased the binding of SIRT1 to the *ACE2* promoter (Clarke et al., 2014). In addition, there is a cAMP-responsive element (CREB)-binding site within an upstream region of the start site containing both p300 (a CREB co-activator that relaxes the chromatin and recruits RNA polymerase II) and the CREB site (Figure 3).

The *in silico* study of candidate binding sites within the 400 bp upstream of the transcription start site identified putative sites for various DNA-binding molecules, with different tissue expression such as CDX2 in the lungs, colon, and terminal ileum; HNF1A in the colon, kidneys, and terminal ileum; FOXA1 in the cervix, colon and terminal ileum; SOX11 in the kidneys, and TCF7/LEF1 in the lungs (Barker and Parkkila, 2020). The *ACE2* promoter also contains an androgen receptor (AR) binding site, and AR antagonists (e.g., enzalutamide, apalutamide) have been reported as being able to decrease SARS-CoV-2 infection (Qiao et al., 2021). Moreover, forkhead box A1 (FOXA1; also known as HNF3α) involved in AR signaling, and bromodomain-containing protein 4 (BRD4) binding sites, overlap with open chromatin regions. Bromodomain and extra terminal domain (BET) antagonists (e.g., JQ1, OTX015), inhibit BRD4, a factor able to interact with positive elongation factor (P-TEFb) cyclin-dependent kinase required for transcription elongation through RNA polymerase II (RNA pol II), also decrease SARS-CoV-2 infection through the inhibition of BRD4. The distal-less homeobox 2 (DLX2) and CCAAT/enhancer binding protein epsilon (CEBPE) are more represented in ACE2-expressing cells (Sherman and Emmer, 2021). Evidence for additional transcription factor binding sites (e.g., SP1, CEBP, GATA3, HNF4A, USF1, etc.) has also been reported (Beacon et al., 2021).

Putative binding sites for signal transducer and activator of transcription, STATs (−662 to −647 region and −911 to −897 region), and interferon-regulatory factors, IRFs, have also been demonstrated (Ziegler et al., 2020). Indeed, interferon modulates ACE2 expression and can lead to the transcription of a truncated form of ACE2, designated as deltaACE2 (*dACE2*) which lacks 356 amino-terminal amino acids and fails to bind to SARS-CoV-2 (Onabajo et al., 2020). The transcription of such a truncated form of ACE2 involves the activation of a promoter located downstream of the transcription start site with a splicing event introducing a new ATG start codon. The analysis of this region identified ISGF-3-, AP-1-, and NF-κB-binding sites (Blume et al., 2021). Treating cells with IFNβ significantly induces the dominant expression of *dACE2* over *ACE2* (Onabajo et al., 2020). In addition, the possible role of alternatively spliced isoforms of ACE2 in SARS-CoV-2 homing, infectivity, and influence on COVID-19 evolution, should be investigated (Heyman et al., 2021; Nikiforuk et al., 2021). Polymorphisms in *ACE2* gene 5′ upstream regions might influence ACE2 expression. Differences greater than 1% of minor allele frequency (MAF) in the 10 Kb region upstream to ACE2 analyzed using data from the 1,000 Genomes project, found 57 polymorphisms (Lanjanian et al., 2021). A single nucleotide polymorphism (SNP), rs5934250, with a change from G to T at approximately 5,700 bp upstream of the start codon of the *ACE2* gene, presented a penetration difference among populations. This allele is almost absent in the East Asian population, while it has a MAF in almost half of Europeans (East Asians: 1%; Africans: 10%; South Asians: 22%; Americans: 29%; and Europeans: 47%). Another SNP, rs2097723, also shows a very heterogeneous distribution among populations (Africans: 7%; South Asians: 22%; Europeans: 28%; Americans: 32%; and East Asians: 42%).

## Human ACE2 polymorphism

Exploration of the *ACE2* genetic polymorphism was conducted to define SNPs associated with hypertension and heart diseases. Special attention was drawn to 14 SNP (rs2285666, rs1978124, rs2074192, rs2106809, rs4830542, rs4240157, rs879922, rs2158083, rs233574, rs1514282, rs1514283, rs4646155, rs4646176, and rs4646188). The best characterized SNP is a splice region variant (rs2285666, G > A, Intron 3/4), known to be associated with hypertension, coronary heart disease, and diabetes (Yang et al., 2015; Pinheiro et al., 2019; Bosso et al., 2020). A number of SNPs, including genotypes of rs2048683, rs233575, rs2158083, rs2074192, rs2106809, rs4240157, rs4646155, and rs4830542 were linked with moderate risks of hypertension, while rs4646188 and rs879922 were linked to high hypertension risks (Yi et al., 2006; Fan et al., 2009; Patnaik et al., 2014; Dai et al., 2015; Meng et al., 2015; Chen et al., 2016; Liu et al., 2018; Luo et al., 2019), and the rs2074192 and rs2106809 were associated with left ventricular hypertrophy in hypertensive patients (Fan et al., 2019). The *ACE2* A_1075_G allele found in China was associated with hypertension and the *ACE2* G_8790_A allele is associated with susceptibility to hypertension, type 2 diabetes, and increased plasma concentration of sACE2 (Niu et al., 2007; Wu et al., 2017; Pinheiro et al., 2019). An allele frequency heterogeneity for the rs2285666 (East Asians: 17%; South Asians: 23%; Americans: 37%; Africans: 48%; Europeans 48%; and with the highest frequency in Indians: 71%) has been reported (Khayat et al., 2020) while the rs4646140 has a MAF ranging from zero in Indians to 13% in Africans. Polymorphisms, including rs233574, rs2074192, and rs4646188 with MAF of 16, 36, and 6%, respectively, were able to induce a significant RNA secondary structure change (Pouladi and Abdolahi, 2021). These alterations may lead to dysregulations in ACE2 transcription/translation or its protein stability. Indeed, in the case of the mutated alleles, the splicing regulatory molecule ETR-3 is unable to bind to the pre-mRNA. Similarly, in the case of the mutated forms of rs2158083 and rs2285666, the binding of YB-1 and hnRNP DL, respectively, are impaired, resulting in exon retention. In the case of the mutated form of rs1514283, the SF2/ASF, and SRp40 proteins bind and lead to the creation of a new intron splicing enhancer and exon inclusion. In the case of the mutated form of rs879922, there is a possibility of interaction with the SC35 and DAZAP1 proteins that leads to exon inclusion. In addition, the binding of proteins of the hnRNP A1, A0, A2/B1, D, and DL family creates a new intronic splice silencer and intron exclusion. In the case of the mutated form of rs4646155, the NOVA-1 protein induces an exon inclusion, while SLM-2 and Sam68 lead to intron exclusion. In the case of the mutated form of rs2106809, the hnRNP H proteins lead to an intron exclusion.

As COVID-19 emerged, it was postulated that SNPs in the *ACE2* gene could affect susceptibility for SARS-CoV-2 infection (Darbani, 2020; Devaux et al., 2020; Hou et al., 2020). Particular attention was paid to the impact of the G_8790_A mutation on the severity of COVID-19, although its role in this disease remains controversial (Gómez et al., 2020; Möhlendick et al., 2021). About 77% of GG genotype, 13% of GA genotype and 9% of AA genotype were found in Caucasian SARS-CoV-2-positive patients and 70% of GG genotype, 14% of GA genotype and 16% of AA genotype carriers in SARS-CoV-2-negative people, respectively. A meta-analysis concluded that the ACE2 variant rs190509934:C (a rare variant) characterized by a lower ACE2 expression in individuals carrying the C allele, reduces the risk of SARS-CoV-2 infection (Horowitz et al., 2021).

**Figure 4:**
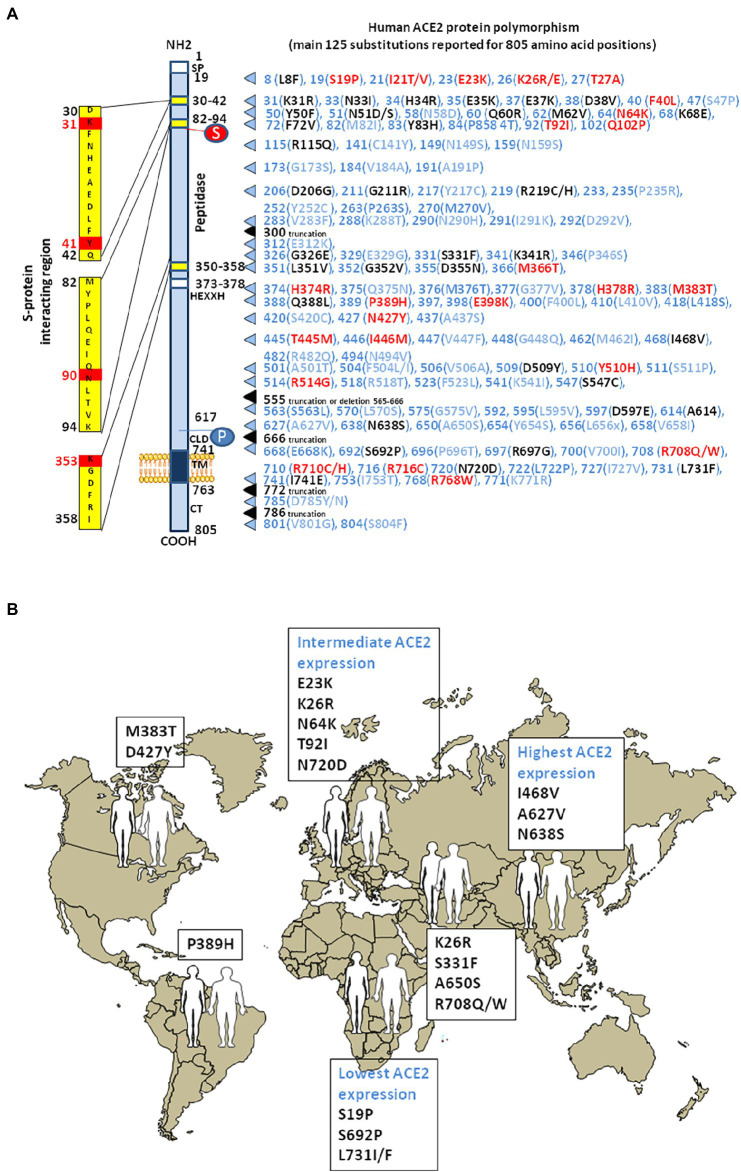
Human ACE2 polymorphism. **(A)** Schematic representation of the cell surface of the human ACE2 molecule and its major domains is drawn on left side of the figure. The amino acid positions are in black. Some of the amino acids considered to be important for viral tropism are marked in red. S, sugar; P, phosphorylation. The right part of the figure is a compilation of the main substitutions described in the literature. To simplify the figure, we used the single letter amino acids code instead of multiple letters code. The ACE2 substitutions in blue are considered neutral. The ACE2 substitutions in red are predicted to increase cell susceptibility to SARS-CoV-2. The ACE2 substitutions in black are predicted to decrease cell susceptibility to SARS-CoV-2. Polymorphisms in intronic regions might modify *ACE2* regulation. Polymorphisms were able to induce a significant RNA secondary structure change. These alterations may lead to dysregulations in ACE2 transcription/translation or its protein stability. **(B)** The main geographical distribution of ACE2 protein polymorphisms in human populations. Representative substitutions in the human mACE2 per geographic areas.

Analysis of inter-individual *ACE2* polymorphism, based on broad genomic databases reveal a link with the susceptibility to SARS-CoV-2 and the severity of COVID-19 (Brest et al., 2020; Cao et al., 2020). The pioneering work by Cao and colleagues identified 15 unique expression quantitative trait loci variants (14 SNPs and 1 InDel) with a higher frequency of minor alleles in the Asian population than in the European population. For example, the rs143695310 variant among East Asian populations was found to be associated with elevated expression of ACE2. Moreover, it was reported that Asian men have a higher ACE2 mRNA expression in their lungs than women, and that Asian people express higher amount of ACE2 than Caucasian and African American populations according to single-cell RNA-seq analysis (Zhao et al., 2020; Figure 4). Similar data were obtained using expression quantitative trait loci (eQTL), indicating a higher expression of ACE2 in South Asian and East Asian populations compared to Europeans, while the lowest expression levels were observed for Africans (Ortiz-Fernández and Sawalha, 2020). Dozen of human *ACE2* variants were identified, which could impact on protein stability (e.g., Lys_26_Arg, Gly_211_Arg, and Asn_720_Asp variants) or internalization (e.g., Leu_351_Val and Pro_389_His variants; Benetti et al., 2020; Cao et al., 2020; Othman et al., 2020). The rs41303171 C polymorphism, which is practically exclusive to Europeans (MAF 1.8%), is a missense SNP causing an Asn_720_Asp replacement, which can trigger a conformational disorder in ACE2 changing viral interactions (Khayat et al., 2020). The Pro_389_His variant occurs in Latino American population with an allele frequency of 0.015%. Only African Americans carry Met_383_Thr and Asp_427_Tyr variants with allele frequencies of 0.003 and 0.01%, respectively. The Arg_514_Gly occurs in African Americans with an allele frequency of 0.003% (Hou et al., 2020). The European population with Arg_708_Trp, Arg_710_Cys, Arg_710_His, or Arg_716_Cys variants in mACE2 may have mild symptom of COVID-19 as ACE2 lose the cleavage site by TMPRSS2 (Hou et al., 2020; Lanjanian et al., 2021). The Ser_19_Pro variant (rs73635825 genotype) common in African populations, may protect against COVID-19 while the Lys_26_Arg variant (rs75548401 genotype) might predispose to severe forms of COVID-19 (Calcagnile et al., 2021). Recently, Suryamohan and colleagues found 298 unique ACE2 variants (Suryamohan et al., 2021). Among these variants they predicted that the Lys_31_Arg polymorphism breaks an interaction with Gln_493_ in the viral RBD and destabilizes the charge-neutralizing interaction with the virus and that the Glu_37_Lys polymorphism disrupts the critical interactions with ACE2 Lys_353_ by removing the polar intramolecular interaction that stabilizes contacts with the SARS-CoV-2 RBD. Similarly, the His_34_Arg was predicted to result in a loss of interface polar contact. Thus, individuals carrying these variants are predicted to be less susceptible to SARS-CoV-2 infection. Fourteen human ACE2 variants (Ile_21_Val, Glu_23_Lys, Lys_26_Arg, Asn_64_Lys, Thr_92_Ile, Gln_102_Pro, Asp_206_Gly, Gly_211_Arg, Arg_219_Cys, Glu_329_Gly, His_378_Arg, Val_447_Phe, Ala_501_Thr, and Asn_720_Asp) which could enhance susceptibility to SARS-CoV-2 were found to have an higher allele frequencies in European populations than East Asian populations, while two additional ACE2 variants (Glu_35_Lys and Phe_72_Val) possibly conferring resistance to the virus, have higher allele frequencies in East Asian populations, while they are low or not expressed in European populations (Chen et al., 2021). Recently, a total of 570 genetic variations (SNP and InDel) on the *ACE2* gene were reported in the Iranian population (Lanjanian et al., 2021).

## ACE2 production and regulation inside human cells

Angiotensin-converting enzyme 2 surface abundance differ among cell types, indicating a complex epigenetic regulation of the *ACE2* gene. The interaction between tissue or cell type specific enhancer/repressor is required for gene expression (Andersson et al., 2014). The *ACE2* gene expression is also increased in individuals with pulmonary arterial hypertension, chronic obstructive pulmonary disease, obesity, diabetes, and older people (Muus et al., 2020; Pinto et al., 2020). In patients with hypertensive cardiopathy a marked ACE upregulation and ACE2 downregulation associated with Ang II/AT1R induced activation of the ERK1/2 and p38 MAP kinase, was reported (Koka et al., 2008). DNA methylation (5mC) was found to be involved in the silencing of *ACE2* gene expression and CpG methylation was greater in patients with hypertension compared to healthy controls (Fan et al., 2017; Chlamydas et al., 2020; Cardenas et al., 2021). In contrast, enhanced ACE2 expression might also be protective in COVID-19 if it increases the peptidase activity of ACE2 thereby reducing Ang II concentration. Hypomethylation of specific sites in the *ACE2* promoter was reported to correlate with increased *ACE2* gene expression (Corley and Ndhlovu, 2020). Three CpGs (cg04013915, cg08559914, and cg03536816) at the *ACE2* gene were reported as having lower methylation in lung epithelial cells compared to the other tissues (Beacon et al., 2021). The search for *ACE2* topologically associating domains (TADs) with active histone markers, including H3 acetylated at K27 (H3K27ac) and H3 trimethylated at K4 (H3K4me3) or repressive histone markers (H3K27me3), revealed the presence of H3K4me3 at the promoter and after the first exon of *ACE2*, and the presence of H3K27ac in human kidneys (Beacon et al., 2021). The association of H3K4me3 correlates with *ACE2* gene expression in the kidneys, heart, and small intestine. In contrast, H3K4me3 peaks are not detected in lung tissues.

**Figure 5:**
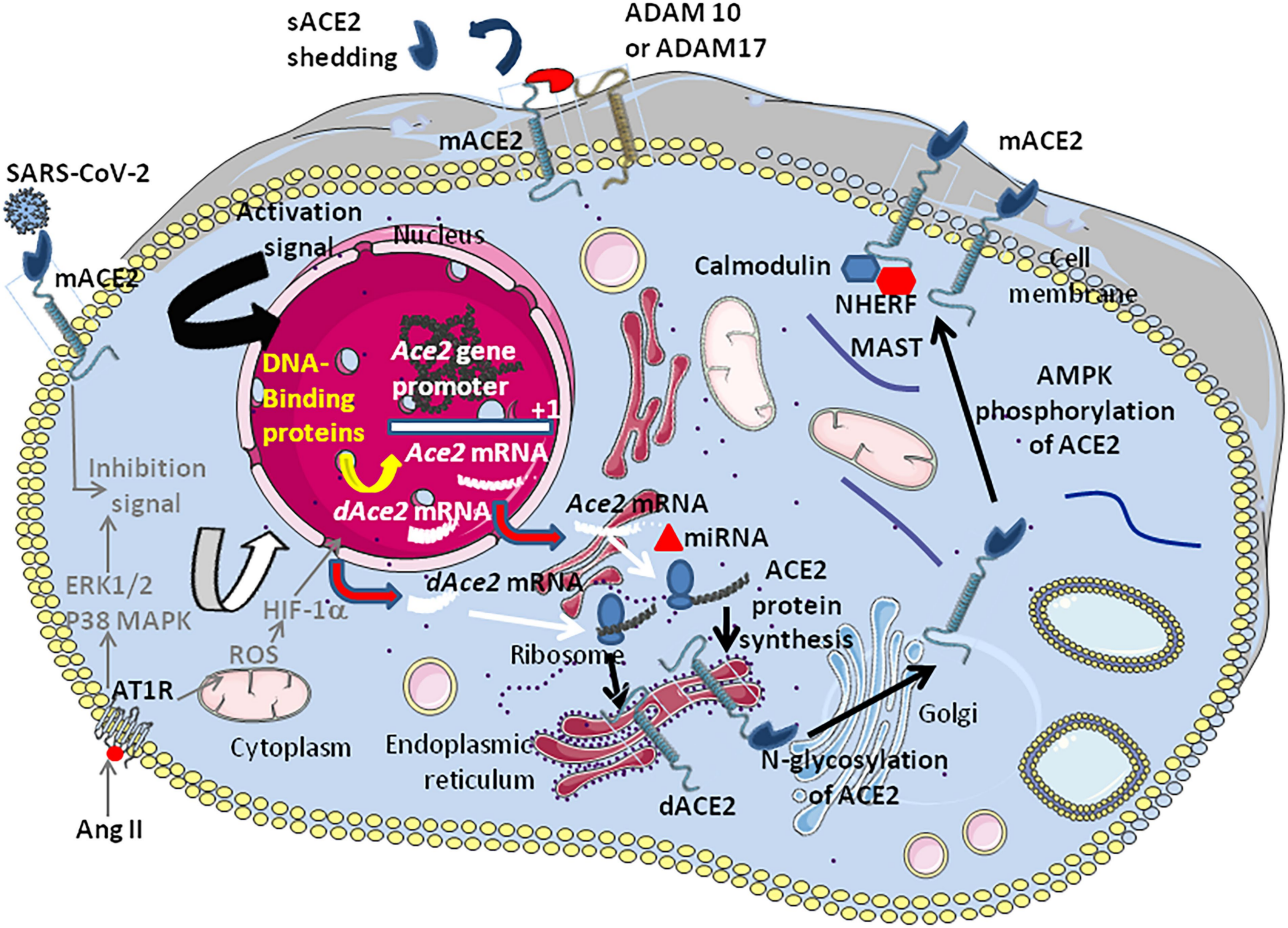
Schematic representation of the regulation of ACE2 expression. The transcription of the *Ace2* gene is under control of several DNA-binding proteins that bind the *Ace2* gene promotor (see Figure 3). In patients with hypertension (hypertensive cardiopathy and hypertensive nephropathy) a down-regulation ACE2 is observed. Angiotensin II was shown to down-regulate ACE2. The inhibition of ACE2 expression was shown to be associated with the activation of ERK and p38 MAP kinases (however this signaling pathway also activates NF-κB which is an activator of *ACE2*, suggesting a complex regulation of positive and negative signals which remains to be characterized). The binding of AngII to AT1R induces the hypoxia-inducible factor (HIF)-1α, which regulates several genes involved in the RAS (e.g., ACE1). Post-transcriptional regulation by miRNA (e.g., miRNA143 and miRNA421) could occur. Following translation the newly synthesized ACE2 proteins are target of post-transcriptional modifications such as phosphorylation of Ser680 by AMPK that enhances the stability of ACE2, and N-glycosylations (seven potential N-glycosylation sites). Once expressed at the cell membrane the mACE2 protein can be regulated by sheddases (e.g., ADAM10 and ADAM17) that cleave the ACE2 extracellular domain and release a circulating soluble form, sACE2. IFNβ is likely to induce dominant expression of *dACE2* over *ACE2*.

MicroRNAs (miRNAs) are non-coding RNAs which can bind the 3′-untranslated regions (3’-UTRs) of target mRNAs, thereby regulating gene expression at a post-transcriptional level. Lysine-specific demethylase 5B, JARID1B, is responsible for the downregulation of several miRNAs that target ACE2 (Henzinger et al., 2020). Putative miRNA-binding sites were identified in the 3′-UTR of the *ACE2* transcript thereby repressing translation. Both the *miR-421*, an miRNA implicated in the development of thrombosis and the miR-*200*c-3p were found to downregulate the ACE2 mRNA expression (Hirano and Murakami, 2020). In contrast the increases ACE2 mRNA expression (Sato et al., 2013; Siddiquee et al., 2013; Zhang et al., 2017). Other miRNAs predicted to bind to ACE2 mRNA 3’-UTR, such as miR-9-5p and miR-218-5p, were found to be differentially expressed in different cell types (Pierce et al., 2020). Moreover, the repression of the Xu and Li, 2021; Figure 5).An *in silico* studies aimed at predicting miRNAs that regulate ACE2-related networks with a possible impact on COVID-19 outcome, suggests that the top miRNAs regulating ACE2 networks are miR-27a-3p, miR-26b-5p, miR-10b-5p, miR-302c-5p, hsa-miR-587, hsa-miR-1305, hsa-miR-200b-3p, hsa-miR-124-3p, and hsa-miR-16-5p (Wicik et al., 2020). sACE2 shed into systemic circulation maintains its ability to generate Ang-(1–7). This process is fine-tuned by ADAM17 (also known as TACE), the metalloprotease ADAM10, and the transmembrane protease serine 2 (TMPRSS2), but only TMPRSS2 increases the entry of both SARS-CoV-1 and SARS-CoV-2 into susceptible cells (Lambert et al., 2005; Heurich et al., 2014; Hoffmann et al., 2020; Qiao et al., 2021). The ADAM17 and ADAM10 sheddases can trigger ACE2 ectodomain shedding by cleavage between amino acids 716 and 741 near the predicted transmembrane domain (Xiao et al., 2014), while TMPRSS2 trigger cleavage between amino acids 697 and 716 (Lanjanian et al., 2021). Phorbol ester and ionomycin as well as the proinflammatory cytokines IL-1β and TNF-alpha, can induce cellular proteases to catalyze sACE2 shedding (Jia et al., 2009). A study of plasma samples from 534 subjects indicated that up to 67% of the phenotypic variation in sACE2 shedding could be accounted for by genetic factors (Rice et al., 2006). mACE2 also interacts with several PDZ-binding proteins such as NHERF, involved in the internalization and recycling of mACE2 (Zhang et al., 2021). The *in silico* study of proteins belonging to the ACE2 interactome and which could be affected by SARS-CoV-2 infection, highlighted that the most affected interactions were associated with microtubule-associated serine and threonine kinase 2 (MAST2), and [Calmodulin 1 (CALM1; Wicik et al., 2020]. It was previously reported that CALM1 inhibitors increase sACE2 shedding by preventing calmodulin binding to the cytoplasmic tail of mACE2 (Lambert et al., 2008).

## ACE2 through the ages

Structural comparisons of genes indicated that *ACE2* and *ACE* arose by duplication from a common ancestor (Riordan, 2003). Although the evolutionary tree of *ACE2* genes from 36 representative vertebrates is consistent with the species evolutionary tree, certain differences found in coelacanths and frogs may suggest a very slow evolutionary rate in the initial evolution of *ACE2* in vertebrates (Lv et al., 2018; Damas et al., 2020; Lam et al., 2020; Luan et al., 2020; Lubbe et al., 2020; Liu et al., 2021). Orthologs of *ACE2* and *ACE* also exist in bacteria, chordates and tunicates, suggesting an early origin of the RAS (Fournier et al., 2012). Although intriguing, the observation that the ACE2-like carbopeptidase from *Paenibacillus* sp. B38 catalyzes the conversion of Ang II to Ang-(1–7) and can suppress Ang II-induced hypertension, cardiac hypertrophy, and fibrosis in mice does not necessarily mean that the origin of the RAS goes back to bacteria but that a molecule with an ACE2-like carbopeptidase activity was maintained during speciation (Minato et al., 2020). ACE2-ancestors may then have acquired important new functions in tissues during speciation, as evidenced in humans. Beside the ACE2-like carbopeptidase, bacteria also express the neutral amino acid transporter SLC6A19, the homologous of B^0^AT1 in human, suggesting that SLC6A19 and the bacterial ACE2 ortholog may have already been molecular partners in bacteria (Gallucio et al., 2020). It is remarkable to note that an ACE-like bacterial protein named *Xc*ACE from *Xanthomonas axonopodis pv. citri*, hydrolyses Ang I into Ang II (Rivière et al., 2007). Other bacteria belonging to *Lactococcus* (*L. lactis*, *L. helveticus*, *L. acidophilus*, and *L. casei*) and *Bifidobacterium* species, release peptides with *in vitro* ACE-inhibitory activity (Fuglsang et al., 2003; Donkor et al., 2007).

The Ance genes from *Drosophila melanogaster* shares similarities with the human *ACE2* (Burnham et al., 2005). In *Acyrthrosiphon pisum*, expression of the insect *ACE2-*ortholog is inducible upon feeding (Wang et al., 2015). The simultaneous KO of *A. pisum ACE2* and *ACE* resulted in enhanced feeding and increased aphid mortality. It was also reported that the challenging of *Anopheles gambiae* with *Staphylococcus aureus* and *Staphylococcus typhimurium* upregulated the transcription of the Anopheles homolog of *ACE*, named AnoACE (Aguilar et al., 2005). Moreover, it was reported that treatment of *A. gambiae* with an ACE inhibitor resulted in larval death (Abu Hasan et al., 2017).

**Figure 6:**
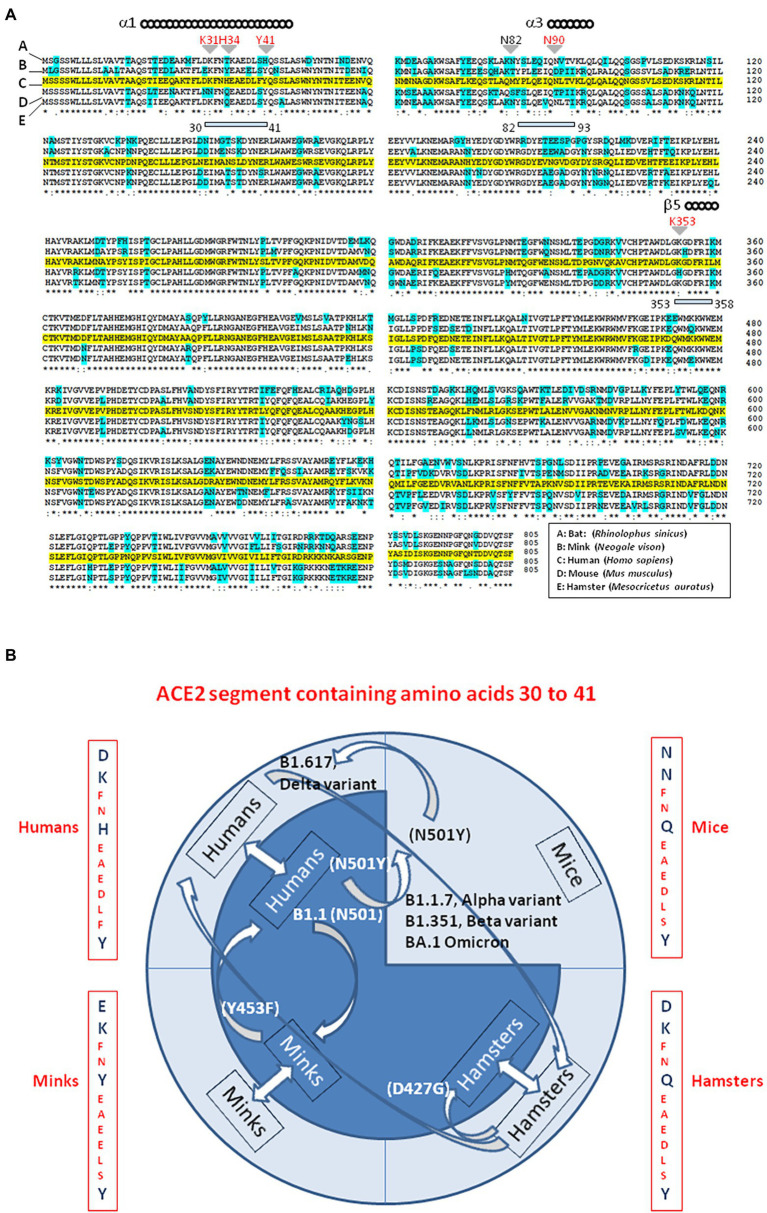
Interspecies viral circulation. **(A)** ACE2 multiple sequence alignment. The consensus ACE2 sequence from humans (*Homo sapiens*; GeneBank BAB40370.1) was compared to ACE2 sequences from mink (Neogale vison NCBI ref. sequence XP_044091953.1), hamsters (*Mesocricetus auratus*; NCBI ref. sequence XP_005074266.1), mice (*Mus musculus*; NCBI ref. sequence NP_081562.2), and bats (*Rhinolophus sinicus*; GeneBank: AGZ48803.1), using the Clustal Omega multiple sequence alignment (EMBL-EBI bioinformatic tool; Copyright © EMBL 2020; https://www.ebi.ac.uk/Tools/msa/clustalo/). The human ACE2 sequence is highlighted in yellow. Amino acids that differ from the human ACE2 sequence in ACE2 from other species are highlighted in cyan. The (*) symbol indicates sequence identity between the ACE2 of the five species. Some of the amino acids found to be important for viral tropism in previous studies (in particular amino acid residues 31, 34, 41, 90, and 353 are important for viral spike binding). **(B)** SARS-CoV-2 is spreading on their ability to recognize a receptor and circumvent the host immune defenses. This principle accounts for the circulation of SARS-CoV-2 between species. Species living in various ecosystem show different amino acid substitutions at positions considered to be required for SARS-CoV-2 spike binding to ACE2. The ACE2 from minks shares 83% amino acid identity with the human ACE2 (63% in the region described to be involved in the interaction with the SARS-CoV-2 spike protein). Despite more than 130 substitutions out of 805 amino acids the interspecies transmission of SARS-CoV-2 from humans to minks and back to humans is possible and generates specific amino acid substitutions in each species, which improved the affinity for the ACE2 receptor as observed in Denmark’ farms. The same applies in the case of the hamster-adapted Delta variant recently described in Hong Kong. SARS-CoV-2 (Wuhan-HU1 strain) cannot use mouse ACE2. It was reported that the B1.1.7 (20I/501Y.V1; UK variant), **(B)** 1.351 (20H/501Y.V2; South Africa variant) and P1 (20J/501Y.V3, Brazilian variant) SARS-CoV-2 variants and other N501Y-carrying variants exhibit extended host range to mice. Moreover, it has been postulated that the new lineage SARS-CoV-2 Omicron (BA.1, BA.2) could have a murine origin. Omicron variants (e.g., BA.5) are the SARS-CoV-2 lineages that currently cause the most cases of human infections. The amino acid differences in residues 30–41 of the N-terminal region of the ACE2 of humans, minks, hamsters, and mice, are indicated. Arrows indicate interspecies circulation of SARS-CoV-2 strains.

While searching for the zoonotic origin of SARS-CoV-2, special attention has been drawn to bats, minks and hamsters ACE2 molecules, as they might serve as viral receptors. Using multiple sequence alignments, we found that the bat ACE2 protein polymorphism grouped in the dendrogram according to the 18 subspecies of bats studied (Devaux et al., 2021c). The ACE2 from *Rhinolophus* bats appeared to be an appropriate candidate for interacting with SARS-CoV-2-related viruses, despite species polymorphism (i.e., *R. sinicus* with Lys_31_, Tyr_41_His, Asn_82_, Asn_90_, and Lys_353_). The Lys_31_Asp variant found in *R. ferrumequinum* may possibly alter the binding of the SARS-CoV-2 spike to the bat mACE2 receptor. The mACE2 sequences from other bat species showed increasing amino acid substitutions at positions considered to be required for SARS-CoV-2 spike binding (e.g., *D. rotundus* with Lys_31_Asn, Tyr_41_, Asn_82_Thr, Asn_90_Asp., and Lys_353_Asn). The mACE2 proteins from *Myotis* bats examined were characterized by Lys_31_Asn, Tyr_41_His, Asn_82_Thr, Asn_90_, and Lys_353_, including substitutions incompatible with SARS-CoV-2-like viruses binding. Regarding the ACE2 from minks we found that the mink ACE2 sequences from *Neovison vison* and *Mustela lutreola* displayed 99.51% similarity to one another, but shared only 83.73 and 83.48% amino acid identity with the human ACE2, respectively (Devaux et al., 2021b). The similarity between human ACE2 and mink ACE2 dropped to 63.34% in the region described to be involved in the interaction with the SARS-CoV-2 spike protein (regions 30–41, 82–93, and 353–358). Despite the fact that more than 130 substitutions out of 805 amino acids were observed between the human ACE2 and mink ACE2 (e.g., 131 substitutions and 133 substitutions for *N. vison* ACE2 and *M. lutreola* ACE2, respectively), including an Asn_90_Asp substitution possibly impacting the affinity of mink ACE2 for the virus, the Lys_31_, Tyr_41_, and Lys_353_ amino acids required for human ACE2 interaction with the SARS-CoV-2 spike protein are conserved in minks mACE2. This amino acids triad is also conserved in hamsters. The Figure 6A, illustrates a comparison of ACE2 amino acid sequences from humans, mink, hamsters, mice and bats.

## ACE2 as SARS-CoV-2 receptor

Severe acute respiratory syndrome coronavirus is an enveloped single-stranded positive-sense RNA virus (its genome contains ∼32 kb). The SARS-CoV-2 viral envelope consists of a lipid bilayer, where the viral membrane (M), envelope (E), and spike (S) structural proteins are anchored. The S proteins surrounding the viral particles consist of two subunits, S1 and S2. This S protein determines the cellular tropism of the virus. In 2020, ACE2 was identified as the main entry receptor for the SARS-CoV-2 virus (Zhao et al., 2020; Zhuang et al., 2020; Baggen et al., 2021). SARS-CoV-2 is the third human coronavirus after SARS-CoV-1 and HCoV-NL63 which use the human mACE2 as a cellular receptor (Li et al., 2003, 2007). A unique feature of SARS-CoV-2 compared with SARS-CoV-1 is the presence of a polybasic motif (RRAR) at the S1/S2 boundary, which can be cleaved by furin (Walls et al., 2020), resulting in a C-terminally exposed RRAR peptide. Two independent studies showed that this peptide directly binds to neuropilin-1 (NRP1) and that NRP1 promotes SARS-CoV-2 infection (Cantuti-Castelvetri et al., 2020; Daly et al., 2020).

A critical step in the SARS-CoV-2 infection cycle is the binding of the homotrimeric viral spike protein through RBD to the peptidase domain of mACE2 (Lan et al., 2020; Shang et al., 2020; Yan et al., 2020). Despite high similarity between the RBD of SARS-CoV-1 and SARS-CoV-2, several amino acid variations in the binding domain of SARS-CoV-2, increase its affinity for ACE2 (Lan et al., 2020; Yan et al., 2020). The interaction is driven by two domains in the S1 subunit of the molecule, namely the RBD and the N-terminal domain (NTD). The NTD displays a flat electropositive ganglioside binding site enabling the virus to interact with lipid rafts of the cell membrane (Fantini et al., 2021). At the N terminus of the viral spike, Gln_498_, Thr_500_, and Asn_501_ of the RBD form a network of H-bonds with Tyr_41_, Gln_42_, Lys_353_, and Arg_357_ of the human mACE2. In addition, in the middle of the bridge, Lys_417_ and Tyr_453_ of the RBD interact with Asp_30_ and His_34_ of ACE2, respectively. Moreover, Gln_474_ of the RBD is H-bonded to Gln_24_ of ACE2, whereas Phe_486_ of the RBD interacts with Met_82_ of ACE2 through van der Waals forces (Yan et al., 2020). Binding of S1 to the mACE2 receptor triggers an ACE2 ectodomain cleavage by ADAM17 (Lambert et al., 2005; Heurich et al., 2014; Oarhe et al., 2015). The ACE2 cleavages by ADAM17 and a serine protease (TMPRSS2 or TMPRSS4) induce the shedding of cellular ACE2 and systemic release of S1/sACE2 complex, and primes for cellular viral entry (Hoffmann et al., 2020). When S1 binds to mACE2, another site on S2 is exposed and cleaved by host proteases. S2 does not interact with mACE2 but harbors the functional elements which guides membrane fusion. So, SARS-CoV-2 can therefore utilize two pathways to infected ACE2 positive cells: the virus can either fuse at the plasma membrane (early pathway) or, it can fuse at the endosomal membrane (late pathway). The privileged pathway is determined by the proteases present at the cell membrane (Wicik et al., 2020; Caillet-Saguy and Wolf, 2021). When the fusion occurs at the cell membrane, this process is followed by the formation of a funnel like structure built by two heptad repeats in the S2 protein in an antiparallel six-helix bundle, facilitating the fusion and release of the viral genome into the cytoplasm. When the protease is absent, SARS-CoV-2 can be endocytosed *via* clathrin-and non-clathrin-mediated internalization and the virion is then activated in endosomal vesicles by the action of low pH-dependant protease Cathepsin L (Tang et al., 2020). Thus, the expression and polymorphism of both ACE2 and TMPRSS2 are likely to dictate SARS-CoV-2 tissue tropism (Hou et al., 2020; Zou et al., 2020). Whether overexpression of mACE2 would facilitate infection (increasing the number of receptors available for the virus) or restrict the risks of developing the most severe forms of the disease, has long been a source of controversy (Vaduganathan et al., 2020). Once bound to mACE2, SARS-CoV-2 down-regulates the cellular expression of the *ACE2* gene and mACE2 protein and the unopposed action of Ang II was deemed responsible for worsening the outcome of COVID-19 (Hendren et al., 2020).

The ACE2 key residues at the ACE2/S-protein-RBD interface include Ser_19_, Gln_24_, Thr_27_, Phe_28_, Asp_30_, Lys_31_, His_34_, Glu_35_, Glu_37_, Asp_38_, Tyr_41_, Gln_42_, Leu_45_, Leu_79_, Met_82_, Tyr_83_, Thr_324_, Gln_325_, Gly_326_, Glu_329_, Asn_330_, Lys_353_, Gly_354_, Asp_355_, Arg_357_, Pro_389_, and Arg_393_ (Suryamohan et al., 2021). The Lys_31_ and Lys_353_ residues in human mACE2 form hydrogen bonds with the main chain of Asn_501_ and Gln_493_ in the RBD. ACE2 variants Ser_19_Pro, Ile_21_Val, Glu_23_Lys, and Lys_26_Arg (which stabilizes core ACE2 α-helical interactions), Thr_27_Ala (which removes interactions between Thr_27_ and Glu_30_), Asn_64_Lys, Thr_92_Ile, Gln_102_Pro and His_378_Arg were predicted to increase cell susceptibility to SARS-CoV-2. In contrast, ACE2 variants Lys_31_Arg (which breaks an interaction with Gln_493_ in the SARS-CoV-2 spike RBD), Asn_33_Ile, His_34_Arg (which results in a loss polar contact at the interface with SARS-CoV-2 spike RBD), Glu_35_Lys (which affects the critical polar contact with SARS-CoV-2 spike Gln_493_), Glu_37_Lys, Asp_38_Val (which compromises the Asp_38_-Lys_353_ interaction), Tyr_50_Phe, Asn_51_Ser, Met_62_Val, Lys_68_Glu, Phe_72_Val, and Tyr_83_His (which prevents insertion of SARS-CoV-2 spike residue Phe_486_ into an hydrophobic pocket driven by residue Tyr_83_), Gly_326_Glu, Gly_352_Val, Asp_355_Asn, Gln_388_Leu, and Asp_509_Tyr were predicted to be less sensitive to SARS-CoV-2 (Procko, 2020; Suryamohan et al., 2021). When considering ACE2 variants, high mACE2 cell-surface expression can mask the effects of impaired binding while low cell surface expression reveals a range of infection efficiencies across variants, supporting a major role for binding avidity during viral entry (Shukla et al., 2021). Using an *in vitro* model of infection of cells expressing suboptimal surface ACE2, it was found that the mACE2 variants Asp_355_Asn, Arg_357_Ala, and Arg_357_Thr abrogated entry of SARS-CoV-2 while Tyr_41_Ala showed only a slight effect on SARS-CoV-2 entry although it inhibited SARS-CoV-1. The NTD and RBD domains in the viral S protein act synergistically to insure virus adhesion (Fantini et al., 2021). Moreover, an inverse correlation was established between ACE2 expression and COVID-19 severity (Chen et al., 2021).

Particular attention was drawn to polymorphism of ACE2 in bat (considered to be a reservoir of SARS-CoV-related virus; Zhou et al., 2020; Wacharapluesadee et al., 2021) this species, and in minks (because they have been shown to be susceptible to infection by SARS-CoV-2 from humans and then to be a source of the virus being able to reinfect humans; Boklund et al., 2021; Oude Munnink et al., 2021; Shuai et al., 2021). It was found that when SARS-CoV-2 of human origin become host-adapted to mink, a Tyr_453_Phe substitution located in the RBD was selected. This process is driven by the fact that mink mACE2 has a Tyr_34_ instead of the H_34_ found in human mACE2 and that the Tyr_453_Phe substitution improves the virus binding to the mink mACE2 (Ren et al., 2021). The hamster is another species of interest for ACE2, because hamster-adapted SARS-CoV-2 Delta variants were isolated in Hong Kong, and the virus was transmitted back to human and further human-to-human transmission was then demonstrated (Kok et al., 2022; Yen et al., 2022). We found that once adapted to the hamster ACE2, the variant virus show mutations (e.g., Asp_427_Gly) that could make this virus more efficient at infecting humans (Fantini et al., 2022). Although a large number of animal species were considered to be susceptible to infection by SARS-CoV-2 (Stawiski et al., 2020), SARS-CoV-2 (Wuhan-HU1 strain) cannot use mouse ACE2 (Zhou et al., 2020). The presence of Asn_30_ (instead of Asp_30_) and Asn_31_ (instead of Lys_31_) in mouse ACE2 is likely to cause the lack of salt bridges and the critical H-bond at the mouseACE2-SARS-COV-2 RBD interface. In addition, the presence of His_353_ (instead of Lys_353_), leads to unfavorable interactions with the SARS-CoV-2 S protein RBD (Brooke and Prischi, 2020; Gao and Zhang, 2020). However, this does not rule out the possibility of low efficiency mouse infection through an alternative receptor. It was reported that the expression of human basigin/CD147 in mice, enabled SARS-CoV-2 infection with detectable viral loads in the lungs (Wang et al., 2020). However, this model remains controversial (Shilts et al., 2021). It has been reported that the B1.1.7 (20I/501Y.V1; United Kingdom variant), B.1.351 (20H/501Y.V2; South Africa variant), and P1 (20J/501Y.V3; Brazilian variant) SARS-CoV-2 variants and other N501Y-carrying variants exhibit extended host ranges to mice (Montagutelli et al., 2021; Shuai et al., 2021). Moreover, it has been postulated that the new lineage SARS-CoV-2 Omicron variant (BA.1, BA.2), has a murine origin (Wei et al., 2021). Indeed, the interspecies conservation of ACE2 turns out to be sufficient to allow viruses that use this receptor to circulate between animal hosts and humans. Viruses do not spread based on species but based on their ability to recognize a receptor and circumvent the host immune defenses. We have proposed that this general principle accounts for the circulation of SARS-CoV-2 between species (Frutos et al., 2021, 2022; Figure 6B).

## Immune response against SARS-CoV-2 and auto-antibodies against ACE2 in COVID-19 patients

Infection with SARS-CoV-2 initiates an antiviral immunoglobulin (Ig)M and IgA response, detectable during the first week of symptoms, whereas IgG are found later. The antibody titres reaches a plateau within 6 days after seroconversion (Guo et al., 2020; Kellam and Barclay, 2020; Long et al., 2020; Zhao et al., 2020). The serum level of SARS-CoV-2 specific IgA is positively correlated with the severity of COVID-19 (Ma et al., 2020; Yu et al., 2020). The state of hyperstimulation of the immune system that occurs in severely ill patients contributes to autoimmune manifestations and is associated with an increased need for oxygen therapy (Gagiannis et al., 2020). Moreover, it was recently reported that Ang II induces ROS release from monocytes able to induce DNA damages and apoptosis in neighboring T-cells leading to lymphopenia in certain patients with severe forms of COVID-19 (Kundura et al., 2022). It is neither the purpose of this paragraph to discuss the complex pattern of immune response in COVID-19 (e.g., a decrease in the total number of CD4+ and CD8+ T cells, B cells, and NK and a recruitment of neutrophils; a massive increase in the release of inflammatory cytokines or ‘cytokine storm’, and chemokines such as IL-2, IL6, IL-7, IL-8, IL-10, TNF, IFN; Amor et al., 2020; Campbell and Kahwash, 2020; Han et al., 2020; Luo et al., 2020; Mehta et al., 2020; Tay et al., 2020; Vitte et al., 2020; Zheng et al., 2020), nor is it to review the abnormal expression of Ag II in COVID-19 patients that could stimulate proinflammatory processes (Naftilan and Oparil, 1978; Moore et al., 2015; Varanat et al., 2017; Silva et al., 2020; Raghavan et al., 2021; Vandestienne et al., 2021; Yamamoto et al., 2021), but rather to briefly summarize the contribution of anti-ACE2 and anti-AT1R auto-antibodies in COVID-19, since these molecules could play an important role in the immunological puzzle of clinical variability of the disease.

**Figure 7:**
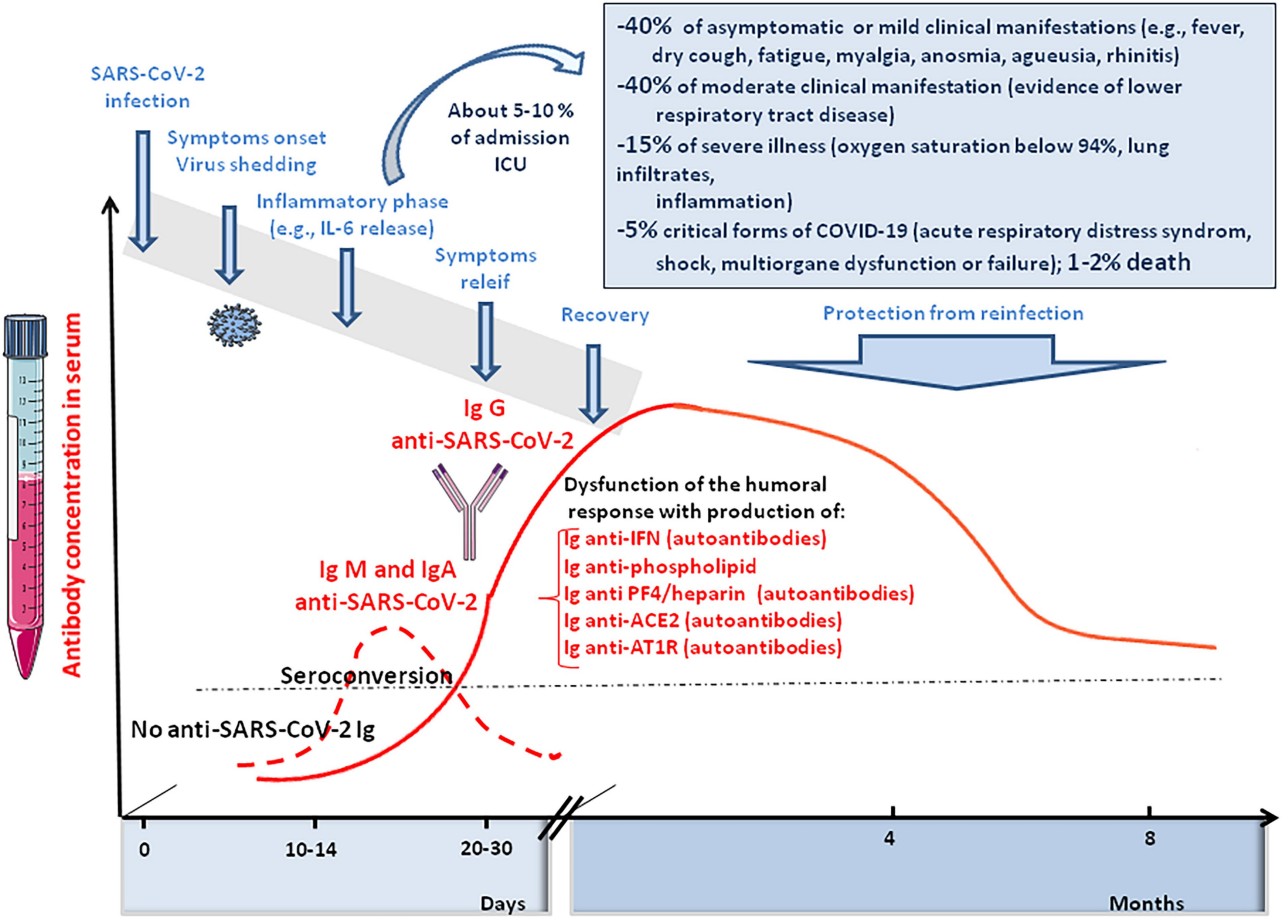
Schematic representation of the clinical course of SARS-CoV-2 infection and COVID-19. It illustrates the immune response of SARS-CoV-2 infected people, including anti-SARS-CoV-2 IgM, IgA, and IgG responses and the induction of auto-immune Ig (anti-phospholipid A, anti-PF4/heparin, anti-ACE2, and anti-AT1R).

What was intriguing in SARS-CoV-2 infected patients with respect to the RAS, was the report of the development of ACE2 auto-antibodies. Among 53 patients who had detectable anti-SARS-CoV-2 RBD, 40 (75%) had anti-ACE2 antibodies (Arthur et al., 2021). Among them, 26 (81%) belonged to the convalescent group and 14 (15; 93%) were patients hospitalized for symptoms of COVID-19. Healthy controls with no history of SARS-CoV-2 were all negative for anti-ACE2 antibodies. The median activity of sACE2 in patients with ACE2 auto-antibodies was 263 pmol/min/ml compared to 1,056 pmol/min/ml for those who did not develop an anti-ACE2 immune response. The binding of anti-ACE2 antibodies to ACE2 in normal cells could have the potential to mediate profound pathophysiological effects long after the original antigen itself has disappeared, particularly in the long term COVID-19 patients (e.g., possibly inducing myocarditis or neurological illnesses; Figure 7).

Considering the similarities between vasculopathy in severe COVID-19 and antibody-mediated rejection after lung transplantation induced by auto-antibodies against AT1R (Cozzi et al., 2017), the presence of AT1R auto-antibodies in COVID-19 patients was investigated and compared to patients with a favorable disease course. A significant increase (42%) of anti-AT1R Ig was found in COVID-19 patients with an unfavorable disease course (Miedema et al., 2021). These AT1R auto-antibodies are expected to mimick the proinflammatory effect of Ang II, as previously reported (Dragun et al., 2005). Tissue transglutaminase (TG2)-mediated modification of AT1R contributes to AT1R auto-antibody production and hypertension associated with preeclampsia; the post-translational modification of Gln_187_ in the second extracellular loop of the AT1R loop creates a neo-epitope that induces the production of an autoantibody that can activate the receptor (Liu et al., 2015). Endothelin receptor type A (ETAR) auto-antibodies were also more frequent in severe COVID-19 patients (Miedema et al., 2021). These antibodies are known to stimulate chemotactic activity and neutrophils trafficking (Cabral-Marques et al., 2018). Both anti-AT1R and anti-ETAR antibodies could be associated with cardiovascular disease and hypertension in severe COVID-19 patients (Philogene et al., 2019).

Among other auto-antibodies found in COVID-19 patients, anti-interferon Ig was found in patients with severe COVID-19 while no such auto-antibodies were found in patients with mild disease (Bastard et al., 2020). Anti-phospholipid antibodies have also been observed as being associated with thrombotic events in COVID-19 cases (Bertin et al., 2020; Daviet et al., 2020; Harzallah et al., 2020; Helms et al., 2020; Manne et al., 2020; Siguret et al., 2020; Tan et al., 2020; Xiao et al., 2020; Zhang Y. et al., 2020; Zuo et al., 2020; Brodard et al., 2021).

## SARS-CoV-2 triggers a vascular and coagulation disease

Venous thromboembolism is a relatively common side effect of SARS-CoV-2 infection. It is characterized by an acute pulmonary embolism or intravascular coagulopathy that predisposes the patients to thrombotic events (Faggiano et al., 2020; Leonard-Lorant et al., 2020; Middeldorp et al., 2020). After the first month of infection, individuals with COVID-19 are at an increased risk of cardiovascular disease, including cerebrovascular disorders, dysrhythmias, ischemic and non-ischemic heart disease, pericarditis, myocarditis, heart failure, and thromboembolic disease (Xie et al., 2022). A nationwide cohort found an increased risk of a deep vein thrombosis up to 3 months after COVID-19, pulmonary embolisms up to 6 months, and bleeding events up to 2 months, with the risk of pulmonary embolism being especially high (Katsoularis et al., 2022). Elevated D-dimers (which reflects the degradation of fibrin and a process of hypercoagulation) upon admission of patients is a marker of hypercoagulation and pulmonary embolism and is associated with increased mortality in severe COVID-19 patients (Lippi and Favaloro, 2020; Sakka et al., 2020; Stefely et al., 2020; Smadja et al., 2021). High levels of D-dimers are found in ∼ 20–40% of critically ill COVID-19 patients (Poissy et al., 2020; Zhang Y. et al., 2020; Zhang S. et al., 2020; Xie et al., 2022). Usual thrombosis prophylaxis is often not sufficient to prevent thrombotic coagulopathy in patients with severe forms of COVID-19 (Berthelot et al., 2020). These lesions usually start with intimal proliferation, followed by fragmented and discontinuous internal elastic lamina (Carvelli et al., 2020; Hofman et al., 2021). Perivascular inflammation was reported to be patchy and scattered, composed mainly of lymphocytes, with thrombi in the branches of the pulmonary artery and focal areas of congestion in the alveolar septal capillaries, as well as septal capillary lesions with wall and luminal fibrin deposition (Deshmukh et al., 2020).

The pathological manifestation of COVID-19 has a strong vascular component, with exacerbated effects on the microvasculature comprising the arterioles, capillaries, venules, and microthrombosis events. The increased occurrence of microvascular thrombi provides a good explanation for the sometimes sudden development of hypoxemia in COVID-19 patients, since the thrombi prevent gas exchange in the oxygenated areas of tissues. Beside the formation of fibrin thrombi, ARDS is characterized by increased alveolar capillary permeability and exudation into the alveoli, where inflammatory cells are present in abundance, as well as coagulation factors including fibrinogen. Regarding COVID-19, it was suggested to name severe pulmonary COVID-19 as “MicroCLOTs” for “microvascular COVID-19 lung vessels obstructive thromboinflammatory syndrome” (Ciceri et al., 2020). The analysis of autopsy lung specimens from COVID-19 patients has shown inflammatory perivascular lymphocyte infiltration, the presence of microvascular thrombi containing platelets, fibrin and numerous neutrophil extracellular traps (NETs) releasing (Carsana et al., 2020; Hofman et al., 2021). Deposits of complement components C3, C4d and C5b-9 were found in the microvasculature of the lungs (Magro et al., 2020). Patients diagnosed with elevated D-dimer and thrombosis during severe forms of COVID-19 have higher blood levels of markers of NETs and calprotectin (Zuo et al., 2020). The formation of NETs in turn, perpetuates complement activation. When activated by proinflammatory cytokines, or NETs, the vascular endothelial cells produce von Willebrand factor (vWF) that retains platelets and leucocytes to the vessel wall and activates coagulation leading to the repair of local damage. Finally, microangiopathic vessel occlusions and endothelium damage has been described in the kidneys (Goshua et al., 2020).

Among the mechanisms implicated in this thrombo-inflammation, AngII seems to have pleiotropic effects. Indeed, regarding the central role played by ACE2 as the viral entry receptor, and its role in the regulation of Ang II blood levels, the balance between ACE2 expression and the accumulation of Ang II in the blood stream may contribute to explain the immunothrombosis. The analysis of RAS dysfunction and Ang II side effects is critical for the understanding of the pathophysiological changes due to SARS-CoV-2 infection. Ang II has a significant effect on the platelet and coagulation/fibrinolytic system and causes mild activation of the coagulation cascade with increases in plasma levels of the thrombin–antithrombin complex and prothrombin (Brown and Vaughan, 2000; Larsson et al., 2000; Fletcher-Sandersjöö and Bellander, 2020; Gando and Wada, 2021). The platelet activation described after COVID-19 is thought to be due in part to the binding of AngII to AT1R. Moreover, SARS-CoV-2 can directly activate platelets by binding to platelet ACE2 (Zhang S. et al., 2020). Through binding to AT1R, Ang II stimulates the expression of Tissue Factor (TF), which triggers coagulation cascade (Nemerson, 1988; Nishimura et al., 1997; Muller et al., 2000; Felmeden et al., 2003; He et al., 2006; Brambilla et al., 2018). Ang II also induces expression of plasminogen activator inhibitor-1(PAI-1), the main inhibitor of tissue plasminogen activator and urokinase-type plasminogen activator, in cultured endothelial cells (Fogari et al., 2011). Increased levels of PAI-1 can occur locally upon SARS-CoV-2 infection, leading to the formation of plugs in the body.

**Figure 8:**
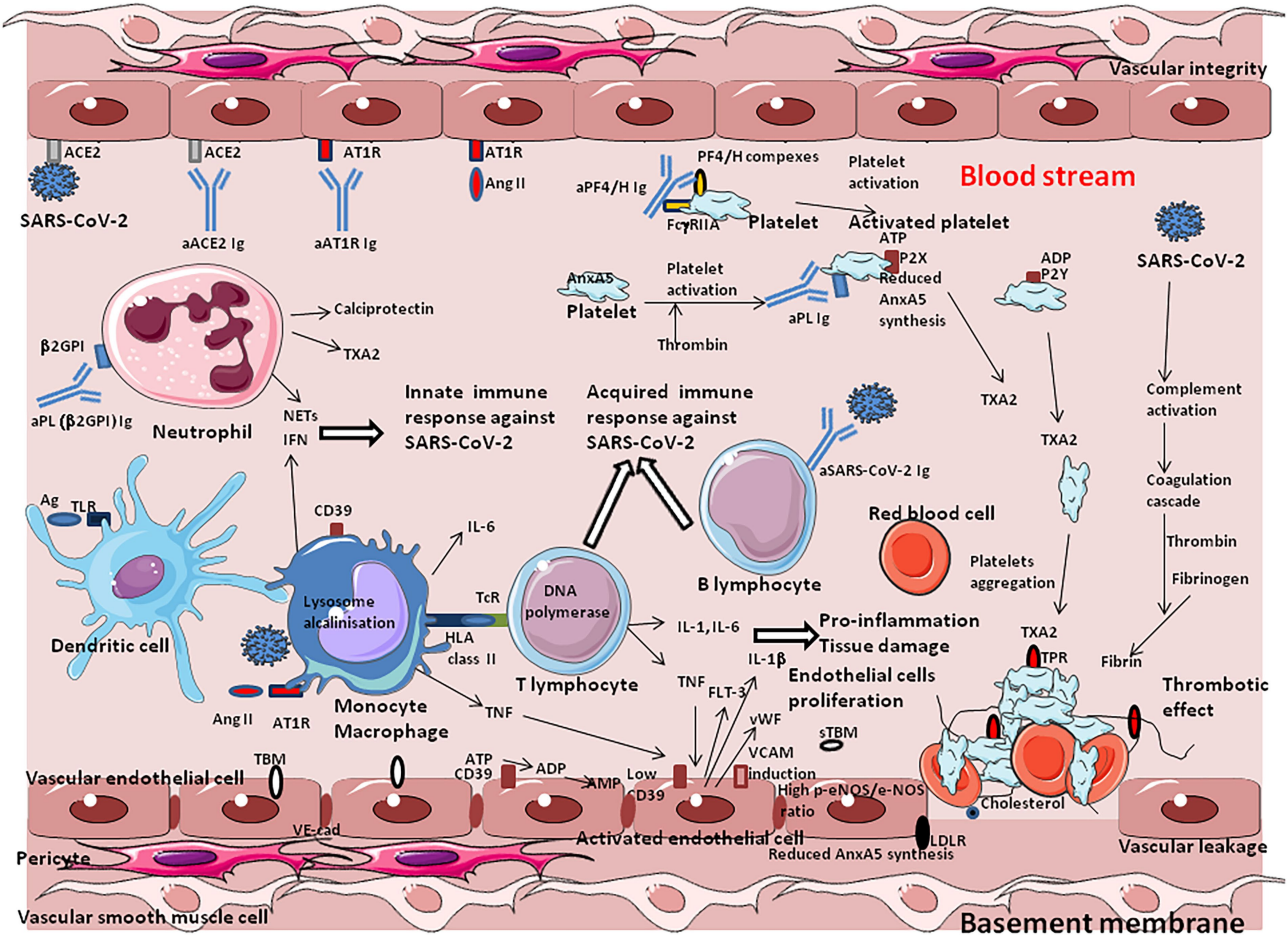
Under physiological conditions, the vascular endothelium, composed of vascular endothelial cells (VECs), functions as an integral barrier with intercellular junctions ensured by adhesion molecules such VE-cadherin. It maintains blood fluidity by acting as an anticoagulant through the suppression of platelet activation and the induction of fibrinolysis, a mechanism including heparan sulfate proteoglycans and CD39. During SARS-CoV-2 infection, innate and acquired immune defense mechanisms are activated with the overproduction of cytokines such as IL-1, IL-2, IL-6, IL-8, IL-17, and TNFα (a phenomenon known as the “cytokine storm”), which disrupts blood vessel walls and provokes tissue damage in the lung parenchyma and the immediately adjacent bronchial alveolar lymphoid tissue. The endothelial cells express the ACE2 molecule that acts as a cell-surface-receptor, facilitating SARS-CoV-2 entry into these cells. The SARS-CoV-2 induced increased concentrations of Ang II have mild platelet-activating effects, thereby enhancing coagulation and are also associated with monocyte and macrophage accumulation, which produces proinflammatory cytokines and worsen hypertension. When blood vessels are strained due to high blood pressure, the endothelial cells of the blood vessels are damaged, and the function of the endothelium at preventing arteriosclerosis is lost. When activated by proinflammatory cytokines, or neutrophil extracellular traps, endothelial cells produce von Willebrand factor, which retains platelets and leucocytes to the vessel wall and activates coagulation systems resulting in the rapid activation of mechanisms leading to the repair of local damage, the accumulation of immune cells to prevent infection, and the aggregation of platelets for primary and secondary hemostasis. Anti-PF4/polyanion (heparin) complex immunoglobulins directly activate platelets *via* their Fc gamma type 2 receptor A (FcγRIIA). The hyper-reaction set up in response to vascular damage, can influence a propensity toward local vascular micro-thrombosis. COVID-19 patients suffer from prominent alveolar oedema, intra-alveolar proteinosis, cell infiltration (including lymphocytes), apoptosis of virally-infected pneumocytes, and fibrin deposition. aPL, antiphospholipids immunoglobulins; aACE2 Ig, anti-ACE2 immunoglobulins; aAT1R Ig, anti-AT1R immunoglobulins; aPF4/H Ig, anti-PF4/heparin immunoglobulins; AnxA5, annexinA5 (or annexin V or anchorin CII; anticoagulant, interact with phospholipids); Ag, antigen; Ang II, angiotensin II; AT1R, angiotensin II receptor type 1; IFN, interferon; CD39/ENTPD1, ectonucleoside triphosphate diphosphohydrolase-1 (also known as P2 receptors: P2X receptors are ion channels that open upon binding of ATP; P2Y receptors mediate cellular response to purine and pyrimidine, such as ATP, ADP, and UTP; in physiological conditions CD39 catalyzes the reduction of ATP and ADP pool to AMP and CD73 transform AMP to adenosine whereas nucleotides released during cell activation/injury bind to P2 receptors to activate thrombo-inflammatory programs); IL-6, interleukin-6; LDLR, low density lipoprotein receptor (bind LDL/cholesterol); NETs, neutrophil extracellular traps; TcR, T-cell receptor; TLR, toll like receptor; TNF, tumor necrosis factor; TXA2, Thromboxane A2 (induce platelets aggregation); TPR, thromboxane A2 prostanoid receptor: VE-cad, VE-cadherin; TBM, thrombomodulin prevents thrombosis; upon endothelial cell activation a soluble form of TBM (sTBM) is released in plasma further promoting procoagulant mechanisms. VWF, von Willebrand factor; Fibrin, fibrin is formed from blood plasma fibrinogen (produced in the liver) by the action of thrombin; red thrombus is composed of erythrocytes enmeshed in a fibrin network.

The binding of SARS-Cov2 to ACE2 at the surface of endothelial cells (ECs) of blood and lymph vessels, leads to activation of the complement system, promoting a pro-coagulative state, leukocyte infiltration, vascular dysfunction, and thrombosis (Jin et al., 2020). In severe COVID-19 patients, the plasma levels intercellular adhesion molecule 1 (I-CAM-1), vascular cell adhesion molecule-1 (VCAM-1), and vascular adhesion protein-1 (VAP-1), are elevated (Escher et al., 2020; Tong et al., 2020), indicating that the endothelial barrier is damaged consecutive to viral infection. Soluble E-selectin, soluble ICAM-1, and soluble platelet endothelial adhesion molecule 1 (sPECAM-1) correlate with disease severity (Li et al., 2021; Vassiliou et al., 2021). ICAM-1 promotes fibrin adhesion and leukocyte transmigration and increased thrombus formation. Disruption of the vascular barrier is associated with the inhibition of protein C, a major anticoagulant (Dahlbäck and Villoutreix, 2005). The plasma levels of vWF, angiopoietin-2, Fms-related tyrosine kinase 3 ligand (FLT-3L), and PAI-1 are significantly elevated in patients with COVID-19 (Liu and Zhang, 2021; Figure 8). Moreover, a decrease ADAMTS13, which ensure vWF hemostatic function, has been reported in severe forms of COVID-19 (Bazzan et al., 2020; Rodriguez Rodriguez et al., 2021). It was also reported that the SARS-CoV-2 main protease M^pro^ causes microvascular brain pathology by cleaving NEMO (an essential modulator of NF-κB) in infected brain ECs (Wenzel et al., 2021).

In response to COVID-19, the activation of ECs was also associated with the overexpression of proangiogenic factors, such as vascular endothelial growth factor (VEGF), basic fibroblast growth factor (FGF-2), and placental growth factors (PlGF; Smadja et al., 2021). Soluble Flt-1 (sFlt-1), a circulating truncated form of the VEGF-A receptor, was markedly increased in severe forms of COVID-19 (Rovas et al., 2020). Damage to ACE2+ pericytes and ECs leads to vascular permeability in severe COVID-19 (Cardot-Leccia et al., 2020; Afzali et al., 2021). The viral Spike induces oxidative stress, ERK1/2 activation through the CD147 receptor and NF-κB nuclear translocation in pericytes, thereby prompting dysfunction of the vascular pericytes (Avolio et al., 2021; Khaddaj-Mallat et al., 2021). CD147, considered to have a potential proatherosclerotic effect (Wang et al., 2015), is upregulated in COVID-19 patients and can act as a receptor for SARS-CoV-2 in cells expressing low ACE2 (Radzikowska et al., 2020). Interestingly, statins, the action of which partly relies on CD147 downregulation, have been recommended in the therapeutic arsenal against COVID-19 (Zhang X.J. et al., 2020).

## Modulation of ACE2 and other actors of the RAS in COVID-19 patients

Coronavirus disease 2019 is a systemic disease characterized by a cytokine storm associated with high levels of C reactive protein (CRP), high fibrinogen, high fibrin degradation to D-Dimers, microvascular injury, and obstructive thrombo-inflammatory syndrome. As knowledge grows, the need for a deeper understanding of the molecular cross-talk leading to thrombosis appears as a research priority in order to gain a better understanding of ARDS and MODS associated with severe COVID-19.

In a pioneer study it was demonstrated that SARS-CoV-1 infection was associated with ACE2 downregulation and impaired degradation of Ang II (Kuba et al., 2005). Since both SARS-CoV-1 and SAR-CoV-2 enter cells through ACE2 and induce simimlar diseases, attention rapidely focused on the consequences of virus-ACE2 interaction on the dysregulation of RAS. This is complexified by the fact that SARS-CoV-2 infection triggers IFN activation, which in turn can upregulate ACE2 (Garvin et al., 2020). Another element of complexity resides in the fact that a greater number of ACE2+ cells seems to circulate in the lungs of patients with severe COVID-19 (Ackermann et al., 2020). Using a swine animal model it was demonstrated that blocking ACE2 (or infusing Ang II) leads to increased pulmonary artery pressure, reduced blood oxygenation, increased coagulation, diffuse alveolar damage, and acute tubular necrosis (Aroor et al., 2016).

**Figure 9:**
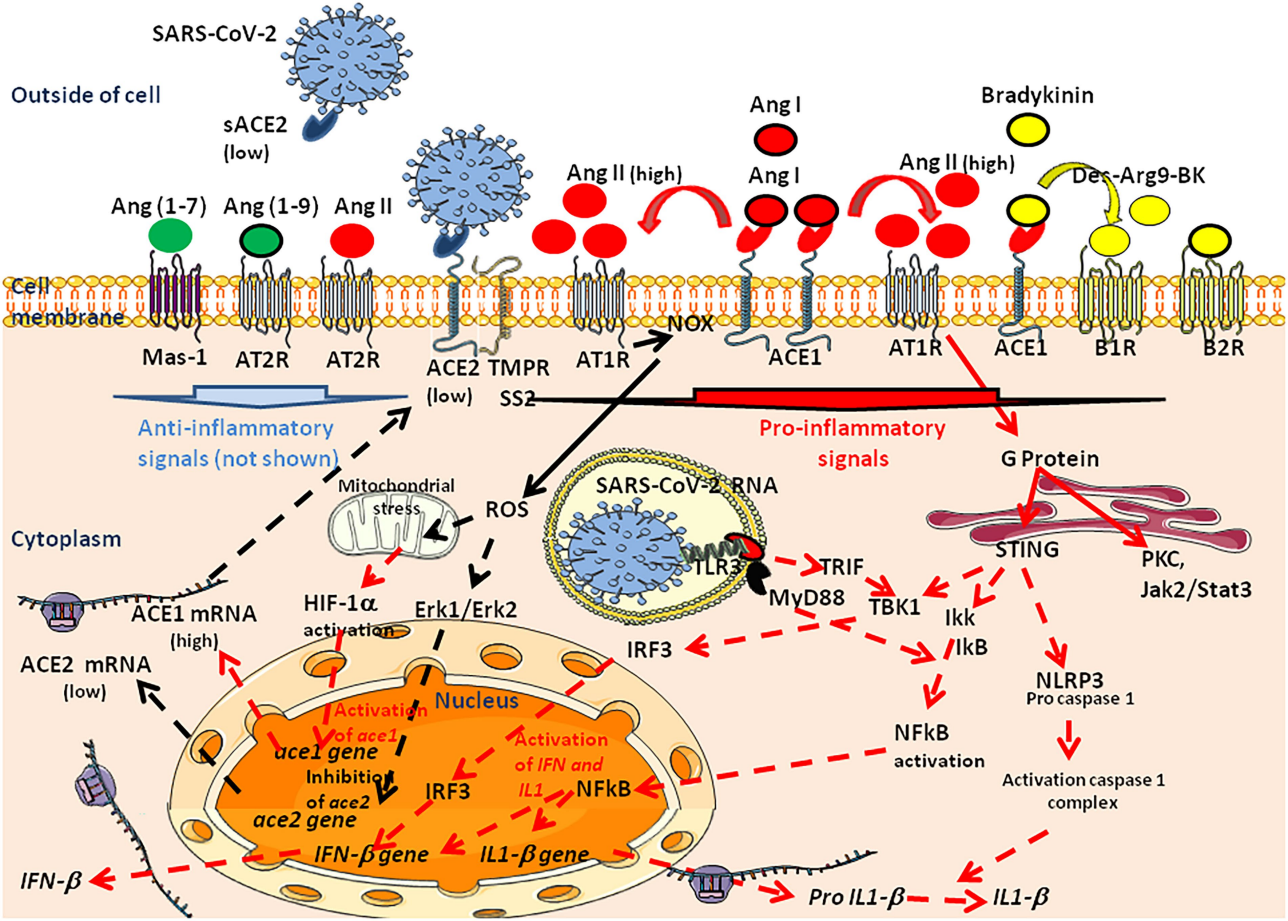
Signaling pathways that are activated during SARS-CoV-2 infection and replication. SARS-CoV-2 infects human cells expressing the ACE2 receptor and the serine protease TMPRSS2. This process results in the downregulation of ACE2 mRNA expression, the reduced expression of mACE2, dysfunction of the RAS and increasing levels of AngII in the circulation. High Ang II levels trigger signaling through AT1R. This activates a number of signaling pathways, such as G protein–mediated (Gq and Gi), Janus kinase/signal transducers and activators of transcription, extracellular signal-regulated kinase (ERK), IFN regulatory factor (IRF)3, NF-κB, NLRP3 procaspase 1 pathways leading to induction of IFNs and pro-inflammatory cytokines, and the HIF-1α pathway. In addition, G protein–independent signaling takes place through the adapter proteins β-arrestin 1 and β-arrestin 2 that can have distinct functional and physiological consequences (not shown). Crosstalk between AT1R and AT2R was evidenced and stimulation of one receptor modulates the expression of the other. Ang II can bind both to AT1R and AT2R, which are receptors with opposite effects but the low expression of AT2R compared to AT1R, account for a privilegied effect of Ang II through AT1R when the plasma levels of Ang II increase. The single AT1R gene in humans encodes a 359-amino-acid protein and AT1R is widely expressed and well conserved between species The MAS receptor can interact with AT1R, explaining that it is the physiological antagonist of Ang II signaling. Mas1 activation induces the second-messenger cAMP, phospholipase A2 pathway, and the phosphoinositide 3-kinase/AKT pathway, and mediates antiapoptotic, anti-inflammatory, vasodilatory, and antithrombotic effects. Excess of Ang II results in organ damage, hypertension, thrombotic microangiopathy, progression to fibrosis, and cardiovascular remodeling.

Despite efforts that have been made to quantify the compound of the RAS in COVID-19 patients, this exploration remained incomplete and debatable. An early study found no difference in the Ang II/Ang I ratio in the plasma sample of 31 COVID-19 patients, but reported that the plasma sACE2 activity was increased in patients treated with an ACE inhibitor (Kintscher et al., 2020). Another study reported increased plasma levels of Ang II in 12 patients with severe COVID-19 pneumonia (Liu et al., 2020). Furthermore, no alteration of RAS was found in a cohort of nonsevere COVID-19 patients (Rieder et al., 2021). More recently, a sevenfold ACE2 increase was found in patients with COVID-19 and Ang II as well as Ang-(1–7) concentrations was significantly higher in patients with severe COVID-19 (Reindl-Schwaighofer et al., 2021). Another investigation in a cohort of 306 COVID-19 patients revealed that elevated plasma sACE2 from COVID-19 patients was significantly associated with severe forms of disease, particularly in hospitalized patients intubated at the time of sample collection (Kragstrup et al., 2021). ARDS in patients with COVID-19 was found associated with an increase in blood pressure and decrease in serum potassium concentration (Vicenzi et al., 2020). Surprisingly, another recent report suggests a significant reduction of Ang II concentration and increased Ang-(1–7) in COVID-19 patients (Martins et al., 2021). The divergent results reported concerning the variation of RAS molecules in plasma from SARS-CoV-2-positive patients could either be explained by the differing severity of COVID-19 in the groups of patients tested and/or by the method used for quantification of the molecules (Figure 9).

Recently, by exploring different biomarkers in a cohort of COVID-19 patients (30 prolonged viral shedders and 14 short viral shedders) we found that circulating blood cells (in particular monocytes) from COVID-19 patients expressed less ACE2 mRNA than cells from healthy volunteers (Osman et al., 2021). Moreover, although we found the expression of sACE2 to be heterogenous among individuals from each group, the sACE2 plasma concentrations were found to be lower in prolonged viral shedders than in healthy controls, while the concentration of sACE2 returned to normal levels in short viral shedders. In the plasma of prolonged viral shedders, we also found higher concentrations of Ang II and Ang I. However, the plasma levels of Ang-(1–7) were found to be almost stable in prolonged viral shedders, but seemed insufficient to prevent the adverse effects of Ang II accumulation, strongly suggesting that increased levels of Ang II contribute to thrombotic events associated with the severe forms of COVID-19.

## Targeting ACE2 for the therapeutic prevention of severe forms of COVID-19

In COVID-19 patients, the downregulation of ACE2 and the reduced capacity to counteract the detrimental effects of Ang II are likely to play a critical role in the development of severe forms of the disease. In experimental animal models, ACE2 KO mice experienced more severe forms of acute lung injury than wild type mice, highlighting the protective role of ACE2 (Imai et al., 2005). The loss of ACE2 resulted in enhanced vascular permeability, neutrophils accumulation and increased lung edema. Both angiotensinogen-specific antisense oligonucleotides and small interfering RNA (siRNA) lowered blood pressure in rat models of hypertension (Mullick et al., 2017; Uijl et al., 2019). In humans with weight excess and hypertension, renin inhibitors (e.g., aliskiren) and ACEi (e.g., ramipril), improve renal and systemic hemodynamics and reduce arterial pressure (Kwakernaak et al., 2017). Thus, therapeutic solutions for reducing COVID-19 severity could be found in the pharmacopeia used by cardiologists to intervene on the RAS.

**Figure 10:**
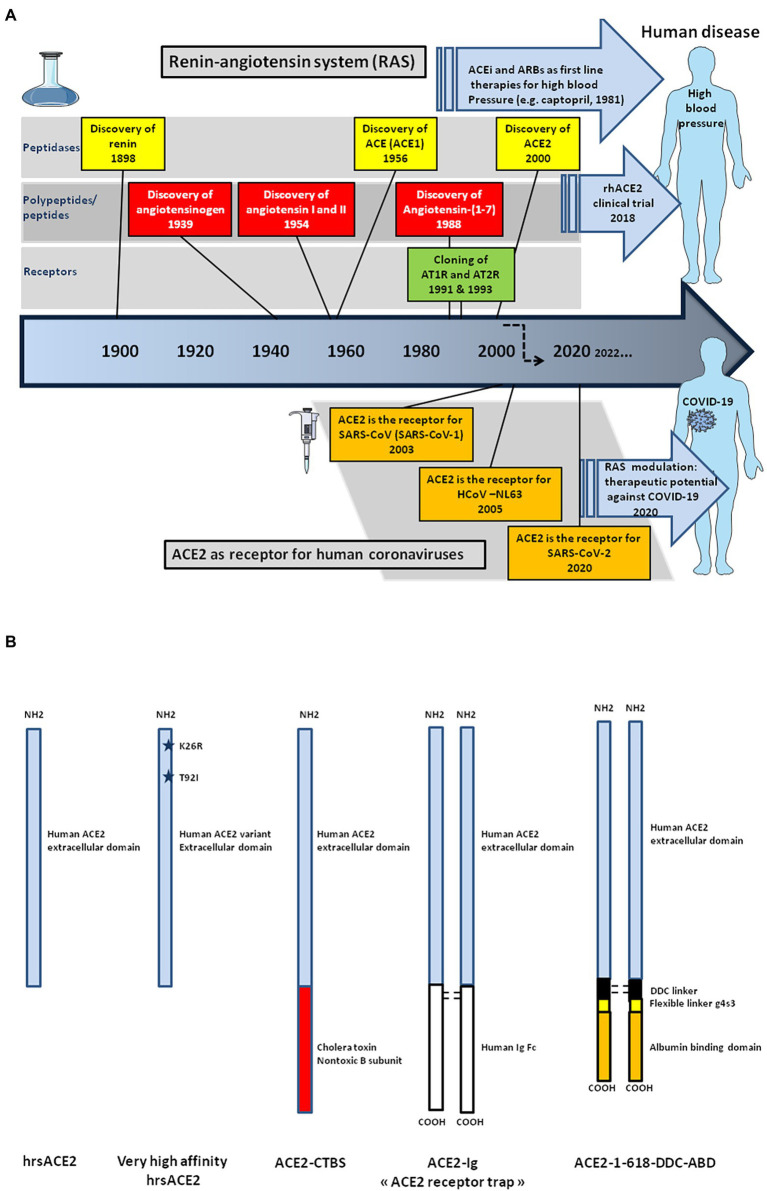
Schematic representation of the historical discovery of the main components of the renin-angiotensin system (RAS) and ACE2 candidate therapeutic molecules. **(A)** In 1898, renin was the first component of the RAS to be discovered. Vasoconstriction of the renal artery was then shown to lead to high blood pressure, thus driving the discovery of hypertensin and angiotonin (a compound later termed angiotensin). Angiotensin was subsequently characterized, as well as two downstream conpounds, the Ang I and Ang II, respectively. The ACE peptidase responsible for processing of Ang I into Ang II was subsequently characterized in 1956. The first orally active angiotensin-converting enzyme inhibitor, captopril, was used as antihypertensive therapy in patients with high blood pressure from the early 80s. The AT1R receptor was cloned in 1991, followed by the cloning of AT2R. Then, the counter-regulatory pathway of RAS was described in 2000, with the discovery of ACE2 by two independent research groups and identification of the Ang-(1–7)/Mas receptor interacting partners was achieved two decades later. The cardioprotective effects of ACE2 were discovered as was its ability to process Ang II into Ang-(1–7). Finally, studies have identified the ACE2 protease domain as the receptor for severe acute respiratory syndrome-coronavirus (SARS-CoV-1) in 2003, HCoV-NL63 in 2005, and, more recently (2020), SARS-CoV-2. **(B)** The ACE2 peptidase extracellular domain known to bind the SARS-CoV-2 Spike can be produced as a recombinant soluble molecule able to neutralize SARS-CoV-2. Various amino acid substitutions can be introduced in the sequence of the ACE2 extracellular domain by genetic engeneering to change the affinity of the recombinant molecule for the viral spike. However, hrsACE2 appears to have a short half-life the efficiency of which could be improved by engineering fusion proteins. The fusion of the rhACE2 extracellular domain with the nontoxic subunit B of cholera toxin (ACE2-CTBS) improves transmucosal transport. The recombinant ACE2-Ig fusion protein consists of a homodimer of the ACE2 extracellular domain linked to an Fc domain of human IgG increasing the stability of the molecule but could also act as cargo for the virus through the Fc-Tag to attach cells like macrophages that express high levels of the Fc receptor. The addition of an albumin binding domain in fusion with ACE2 extend the duration of ACE2 action.

All FDA approved drugs for treatment of patients with high blood pressure (renin inhibitors, ACEi, and ARBs) are primarily designed to block or reduce the detrimental effects of Ang II (Wright, 2000; Mentz et al., 2013; Arendse et al., 2019; Figure 10A). Reducing the formation of Ang II by ACEi or antagonizing its effect by blocking the AT1R through ARBs may be a suitable strategy for reducing symptoms of COVID-19 patients (Schiffrin et al., 2020). ARBs (e.g., losartan) were found to protect against acute lung injury through the reduction of Ang II/AT1R stimulation (Shen et al., 2009; Meng et al., 2020). Hypertensive patients taking ARBs presented a lower risk of severe COVID-19 (Sarzani et al., 2020). However, the interpretation of the benefits and harmful effects of ACEi and ARBs may be premature due to the multiple effects of such molecules on the RAS (Kai et al., 2021; Tereshchenko et al., 2022). Indeed, it has been reported that ACEi and ARBs increase ACE2 (Ferrario et al., 2005; Furuhashi et al., 2015), which could also increase the binding of SARS-CoV-2. However, it has been reported that ACE2 is not increased by ACEi or ARBS in the respiratory cilia (Lee et al., 2020). We recently reported that *in vitro* treatment of SARS-CoV-2 permissive ACE2+/AT1R+ Vero E6 cells with various ARBs resulted into ∼50% increase in SARS-CoV-2 production correlated with the ARBs-induced up-regulation of ACE2 expression (Pires de Souza et al., 2022). However, we also observed a downregulation of AT1R, suggesting that Ang II harmful effects should be strongly reduced (Pires de Souza et al., 2022). The upregulation of ACE2 can have opposed effects on SARS-CoV-2 infection and organ pathophysiology (Devaux, 2020). The study of large cohorts support the beneficial effects of RAS inhibitors in patients with COVID-19 (Bean et al., 2020; Zhang P. et al., 2020). In patients, ARBs is preferred over ACEi for first line hypertension treatment and discontinuing treatment is not required (Abbasi, 2021; Lopez et al., 2021). Since, the activation of AT1R by Ang II induces ROS through the NADPH oxidase pathway and activates the hypoxia-inducible factor (HIF)-1α leading to the synthesis of the transient receptor potential channel ankyrin repeat (TRPA1) which controls intracellular calcium increase and potentially contributes to pulmonary inflammation, it was also suggested to use calcium channel blokers as an alternative to ACEi and ARBS (Fang and Karakiulakis, 2020; Tignarelli et al., 2020; Devaux and Raoult, 2022; Zhang et al., 2022). Moreover, acting on HIF-1α, may improve the outcome of COVID-19 by decreasing hypoxia (Devaux and Raoult, 2022).

*In silico* methods of molecular docking have been used to develop allosteric activators of ACE2 such as xanthenone (XNT), the antiprotozoal dimiazene aceturate (DIZE) drug, and resorcinolnaphthalein (Hernández Prada et al., 2008; Gjymishka et al., 2010). Activation of ACE2 by XNT, prevented elevated right ventricular systolic pressure, ventricular hypertrophy, and increased pulmonary vessel wall thickness (Ferreira et al., 2009; Fraga-Silva Rodrigo et al., 2013; Cole-Jeffrey et al., 2015). DIZE was found to reduce the severity of hyperoxic lungs injury by inhibiting the inflammatory response and oxidative stress, to attenuate the myocardial infarction, and to prevent atherosclerosis by increasing ACE2 mRNA expression (Kulemina and Ostrov, 2011; Qi et al., 2013; Shenoy et al., 2013; Haber et al., 2014; Qiu et al., 2014; Goru et al., 2017; Fang et al., 2019; Qaradakhi and Gadanec, 2020). Various FDA-approved molecules are under study for evaluating their ability to reduce COVID-19 severity (Albini et al., 2020; Lubbe et al., 2020; Qaradakhi and Gadanec, 2020). For example, the virtual screening of 2,456 approved drugs as inhibitors of SARS-CoV-2 spike-ACE2 interaction highlighted the properties of riboflavin (a vitamin), fenoterol (a bronchodilator), vidaranine (an anti-neoplastic agent), and cangrelor (an anti-platelet agent; Prajapat et al., 2020).

A better understanding of the role of the ACE2, encouraged the use of human recombinant soluble ACE2 (hrsACE2) in medicine (Kuba et al., 2005; Oudit et al., 2010; Johnson et al., 2011). In a tolerability study in healthy volunteers, doses up to 1.2 mg/kg hrsACE2 were administered intraveinously and the plasma half-life of the molecule was in the range of 10 h (Haschke et al., 2013). Despite a fast clearance rate, the administration of hrsACE2 alleviated the severity of influenza A H7N9 and respiratory syncytial virus (RSV)-induced lung injury (Yang et al., 2014; Gu et al., 2016). This hrsACE2 was also found to reduce IL-6 when given to healthy volunteers suffering from ARDS (Khan et al., 2017; Zhang and Baker, 2017). Through its binding to the viral S protein, sACE2 could act as a decoy receptor and could reduce the harmful effect of Ang II by making mACE2 available for the conversion of Ang II (Patel et al., 2014; Devaux et al., 2020; Issa et al., 2021; Krishnamurthy et al., 2021). The infusion of a single dose of hrsACE2 (GSK2586881 at 0.4 mg/kg *i.v.*), was found to be well-tolerated and to have potential haemodynamic benefits in pulmonary arterial hypertension (Hemnes et al., 2018). A hrsACE2 clinical trial is ongoing (NCT00886353) for the treatment of cardiovascular diseases (Ghatage et al., 2021). It has been reported that ACE2 S_680_D gain-function knock-in mice are protected against hypoxia-induced pulmonary hypertension (Zhang et al., 2018). This has also opened the way to modified *ACE2* for gene transfer (Guignabert et al., 2018). It was recently demonstrated that recombinant ACE2 is effective for treating SARS-CoV-2 RBD protein-aggravated LPS-induced acute lung injury in a mouse experimental model and that the protection occurs by acting on the ACE2-AngII-AT1R-NOX1/2 axis that is otherwise overactivated by the SARS-CoV-2 infection (Zhang et al., 2022).

A proof-of-concept of the efficiency of the hrsACE2 therapeutic approach in COVID-19 was described in a case report of a 45-year-old woman infected by SARS-CoV-2 who was admitted to hospital with a 7-day history of severe symptoms. Two days after hospital admission, she was treated with 0.4 mg/kg of hrsACE2 intravenous infusion twice daily. Surprisingly after the first injection the patient became afebrile, the biological investigation indicated a marked reduction of Ang II, an increase of sACE2 in plasma, and her clinical condition improved gradually (Zoufaly et al., 2020). A large phase II clinical trial has been initiated by the Austrian pharmaceutical company APEIRON to treat COVID-19 patients with APN01-rhACE2. More recently, hrsACE2, in combination with sub-toxic remdesivir, was found to reduce viral load by 60% in a model of SARS-CoV-2 infected kidney organoids (Monteil et al., 2021).

Given such encouraging results, it seemed important to design molecules with improved activity against SARS-CoV-2 (Maiti, 2021; Figure 10B) A fusion molecule consisting of murine rACE2 with a Fc fragment (rACE2-Fc), demonstrated a long-lasting ability to protect organs in mice models of Ang II-dependent hypertension (Liu et al., 2018). It was also reported that *Lactobacillus paracasi* probiotic expressing a hrACE2 extracellular domain in fusion with the nontoxic subunit B of cholera toxin, resulted in increased ACE2 activities in serum of mice treated with this compound (Verma et al., 2019). Another molecule, rACE2 extracellular domain fused to the FC region of the IgG1, has been shown to neutralize viruses pseudotyped with SARS-CoV-2 spike proteins *in vitro* (Lei et al., 2020). A new set of molecules named “ACE2 receptor trap” that contain the extracellular domain residues 18–614 (including the SARS-CoV-2 RBD), and collectrin domain of ACE2 fused to human IgG1 Fc fragment for increased stabilization and avidity, were designed (Glasgow et al., 2020). *In silico*, ACE2 variants Lys_26_Arg and Thr_92_Ile were predicted to have increased affinity for the viral S protein when compared to wildtype ACE2. Consistent with this, soluble ACE2 Lys_26_Arg and Thr_92_Ile were more effective in blocking the entry of the SARS-CoV-2 S protein pseudotyped virus (Suryamohan et al., 2021). Another type of therapeutic molecules containging the hrsACE2 fused with a 5 kD albumin binding domain and bridged *via* a dimerization hinge-like peptide motif (termed ACE2 1-618-DDC-ABD) was first tested in an animal model prevented mortality in the treated group while untreated animals became severely hill and were found to have extensive pulmonary hemorrhage and mononuclear infiltrates (Hassler et al., 2021). The very rapid accumulation of three-dimensional structural data is likely to greatly accelerate the development of molecules aimed at treating COVID-19 patients (Sorokina et al., 2020).

## Discussion

For virologists, ACE2 is the receptor for Sarbecoviruses. But to see ACE2 as a simple receptor necessary to initiate the replication cycle of the virus would be to ignore the essential role of ACE2 in the pathophysiology of COVID-19. ACE2 was not maintained during species evolution to wait for an unlikely meeting with a spike of Sarbecovirus. The regulatory function of the RAS pathway naturally devolved to ACE2 is the key element to be considered. The imbalance of the RAS pathway followed by the uncontrolled elevation of Ang II levels in SARS-CoV-2 infected patients and signaling through AT1R is the triggering event that can lead to severe forms of COVID-19. Thereby, COVID-19 is primarily a vascular rather than a respiratory disease and Ang II/AT1R blockade might attenuate progression to COVID-19.

The global COVID-19 Host Genetics Initiative (HGI) was set up to bring together international experts in human genetics and epidemiology to explore the genetic determinants of COVID-19 susceptibility, who have shed light on several host factors including ACE2, ACE, TMPRSS2, several chemokine receptors, the IL-6 receptor, IFN, and HLA, which are likely at the forefront of parameters affecting the disease severity (Correale et al., 2020; Ellinghaus et al., 2020; Initiative, 2020; Karaderi et al., 2020; Lorente et al., 2020; Nguyen et al., 2020; Strafella et al., 2020; Zhang Q. et al., 2020; Fricke-Galindo, 2021; Goujon et al., 2021; Martin-Sanchos et al., 2021; Pairo-Castineira et al., 2021). The list of genes possibly involved in the severity of COVID-19 continues to grow and indicates that the predisposition to severe COVID-19 is multifactorial. Understanding these pathways may help identifying targets for COVID-19 therapy and prophylaxis. Although the implication of a multiplicity of genes in the severity of COVID-19 is oubvious when considering the heterogenity in patient’s cases, we consider members of RAS as the main actors of the pathophysiological process. Besides ACE2 and Ang II, RAS can also be regulated by insertion/deletion (I/D) polymorphism of the ACE gene increasing the risk of severe forms of COVID-19 (Marshall et al., 2002; Harrap et al., 2003; Sayed-Tabatabaei et al., 2006; Gupta et al., 2009; Yamamoto et al., 2021). Association between the I/D polymorphism and blood pressure status has been reported (Jeunemaitre et al., 1992; Schmidt et al., 1993; Duru et al., 1994; Kario et al., 1999; Giner et al., 2000; Martinez et al., 2000; Agachan et al., 2003). Infusion of Ang I into normosensitive men was followed by higher venous levels of Ang II and increase in blood pressure in D/D carriers compared with I/I carriers (Ueda et al., 1995). Moreover, it has been reported that higher plasma IL-6 levels can be detected in ST segment elevation myocardial infarction patients, when the D allele is present (Dai et al., 2019). The possible association between the ACE genotype and the severity of COVID-19 should be further explored (Celik et al., 2021; Verma et al., 2021). One report indicates that the prevalence of D/D polymorphism is higher in COVID-19 patients with pulmonary embolism (PE) than patients without PE (Calabrese et al., 2021).

Our review highlights that the most important factors associated with severe COVID-19 outcome are related to the RAS and the regulation of blood pressure and coagulation. By focusing our attention to ACE2, we have come to the conclusion that this molecule may potentially play contrasting roles at different stages of the disease, with its ability to enable viral entry into the cell at early stages of infection thereby increasing disease susceptibility and later by decreasing Ang II/AT1R signaling thereby reducing the severity of the disease. Revisiting the structure and function of this molecule highlights the crucial role of ACE2 in the pathophysiology of sarbecoviruses-induced diseases, particularly in the context of inflammation and thrombosis. A low expression of ACE2 in the respiratory tract (e.g., epithelial cells, arterial and venous endothelial cells present in abundance in the lungs, and arterial smooth muscles) is associated with increased circulating levels of Ang II. The interaction of Ang II with AT1R and activation of various AT1R-dependent signaling pathways induce ROS release from monocytes able to trigger DNA damages and apoptosis in neighboring T-cells leading to lymphopenia, and endothelial injury by inhibiting NO synthesis. It is associated with vasoconstriction, hypertension, vascular permeability, fluid extravasation, and accelerated thrombosis in arterioles by activating hemostasis and the complement system. This process is accompanied by a recruitment of neutrophils and macrophages to the affected tissues leading to the “cytokine storm” (e.g., IL-6, MIP2, TNFα, and IFN responses). Perivascular inflammation is composed mainly of lymphocytes, with thrombi in the branches of the pulmonary artery and focal areas of congestion in the alveolar septal capillaries, as well as septal capillary lesions with wall and luminal fibrin deposition. Taken as a whole, these observations lead us to assume that instead of considering COVID-19 as respiratory tract diseases, we should rather see this disease as a clinical picture of hypercoagulopathy, microvascular immunothrombosis, and hyperinflammation. The loss of ACE2 function after the binding of SARS-CoV-2 is driven by mACE2 receptor endocytosis, activation of proteolytic cleavage of mACE2 and *ACE2* gene transcriptional downregulation. Accurate quantification of RAS biomarkers should be added to the collection of tools aimed at monitoring COVID-19 infection both at pre-clinical and clinical levels.

According to the literature and our own observations, ACE2 and Ang II are the most relevant host factors in later stages of the disease and ACE2 should be seen as an ally in the global fight against COVID-19 and should be considered when designing appropriated drugs for COVID-19 therapy. It now appears that we can see the direction in wich work to deal with this disease should head. It requires treatment consisting in maintaining the homeostasis of the RAS pathway by preventing the elevation of the circulating levels of Ang II through sufficient biodisponibility of ACE2 to hydrolyze Ang II (including the use of human recombinant soluble ACE2), by inhibiting the Ang II/AT1R axis using ARBs which decrease the surface expression of AT1R, and/or by using calcium channel blokers as an alternative to ACEi and ARBS.

## Author contributions

CD conceived the manuscript and wrote the first draft. LC-J participated in the correction of the manuscript. Both authors contributed to the article and approved the submitted version.

## Funding

This work was supported by the French Government under the “Investissements d’avenir” (Investments for the Future) program managed by the Agence Nationale de la Recherche (French ANR: National Agency for Research; reference: Méditerranée Infection 10-IAHU-03 to Professor Didier Raoult), and annual funds from Aix-Marseille university and IRD to the MEPHI research unit (Director: Professor Jean-Christophe Lagier). Other funding sources were limited to the salaries of the authors (Center National de la Recherche Scientifique for CAD, Assistance Publique Hôpitaux de Marseille for LCJ), with no other role or involvement.

## Conflict of interest

CCD declares a link of interest with the Sanofi and Merck pharmaceutical companies.

The remaining author declares that the research was conducted in the absence of any commercial or financial relationships that could be construed as a potential conflict of interest.

## Publisher’s note

All claims expressed in this article are solely those of the authors and do not necessarily represent those of their affiliated organizations, or those of the publisher, the editors and the reviewers. Any product that may be evaluated in this article, or claim that may be made by its manufacturer, is not guaranteed or endorsed by the publisher.
